# Supplementary material for: Identification of Faecalibacterium prausnitzii strains for gut microbiome-based intervention in Alzheimer’s-type dementia
Source: Cell Rep Med. 2021 Sep 14;2(9):100398. doi: 10.1016/j.xcrm.2021.100398 (PMC8484692; doi:10.1016/j.xcrm.2021.100398)
Supplement: Document S1. Figures S1–S6 and Tables S1 and S2 [file mmc1.pdf]

Cell Reports Medicine, Volume *2*

Supplemental information

Identification of *Faecalibacterium prausnitzii* strains for gut microbiome-based intervention in Alzheimer's-type dementia

Atsushi Ueda, Shoji Shinkai, Hirotsugu Shiroma, Yu Taniguchi, Sayaka Tsuchida, Takahiro Kariya, Tomohiro Kawahara, Yodai Kobayashi, Noriyuki Kohda, Kazunari Ushida, Akihiko Kitamura, and Takuji Yamada

Supplemental information:

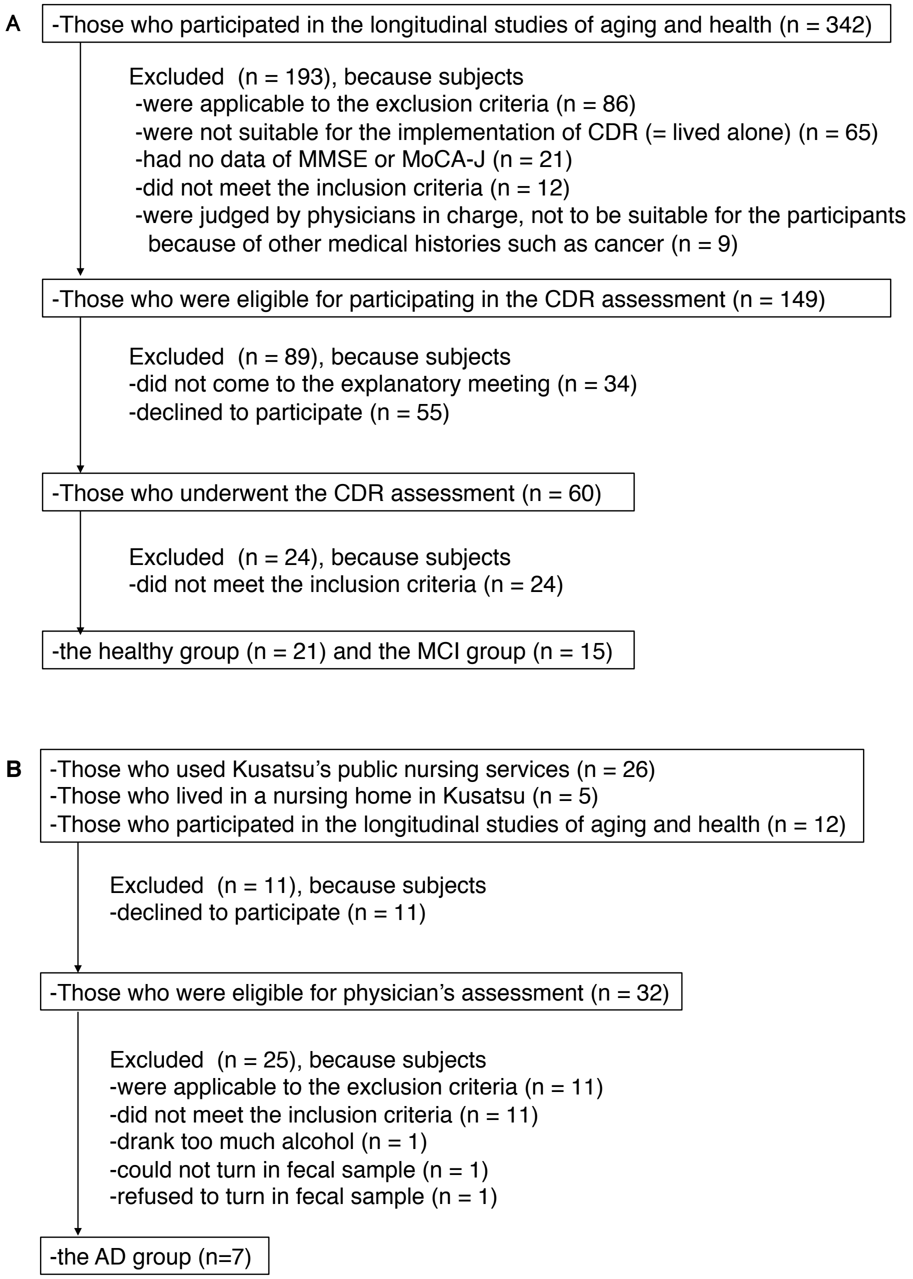

Figure S1. Flow diagram of the selection of the subjects. Related to Figure 2.

(A) Flow diagram showing the selection of subjects in the healthy group and the MCI group. (B) Flow diagram showing the selection of subjects in the AD group.

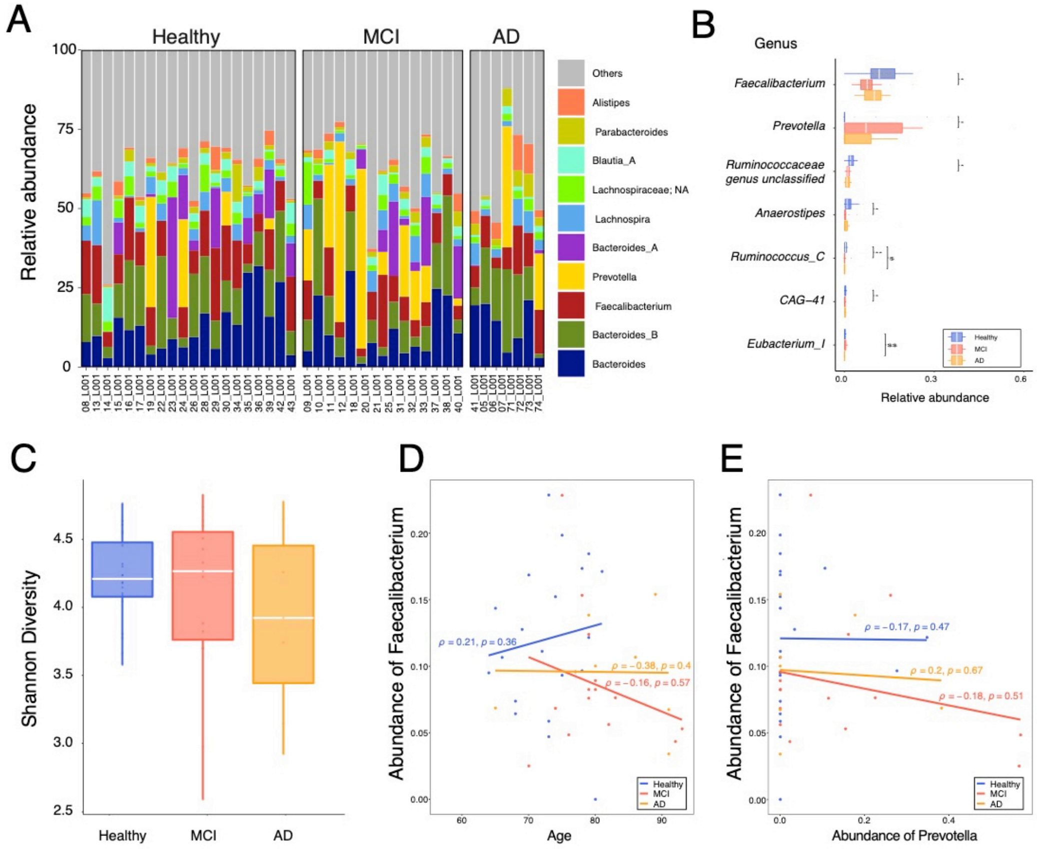

Figure S2. Plot showing the relative abundance of genera, alpha-diversity, and the correlation between the relative abundance of *F. prausnitzii* and age or the relative abundance of *Prevotella*. Related to Figure 2 and Table S4.

(A) Stacked bar plot showing the overall genus composition in each individual. (B) Box plot showing the interquartile range (IQR) of the relative abundance of genera. Seven genera whose abundance was significantly different either between the healthy (n=20) and the MCI group (n=15) or between the healthy and the AD group (n=7) are shown (Wilcoxon rank sum test, the healthy vs the MCI: $^{**}p < 0.01$, $^{*}p < 0.05$, the healthy vs the AD: $^{\$\$}p < 0.01$, $^{\$}p < 0.05$). (C) Box plot showing the IQR of the Shannon-Wiener alpha-diversity index, which did not differ between groups. (D, E) Scatter plot showing Spearman's ρ and p value between the relative abundance of *F. prausnitzii* and age or the relative abundance of *Prevotella* of the healthy (blue), the MCI (red), and the AD group (orange). There was no significant correlation between the abundance of *Faecalibacterium* and age or the abundance of *Prevotella*.

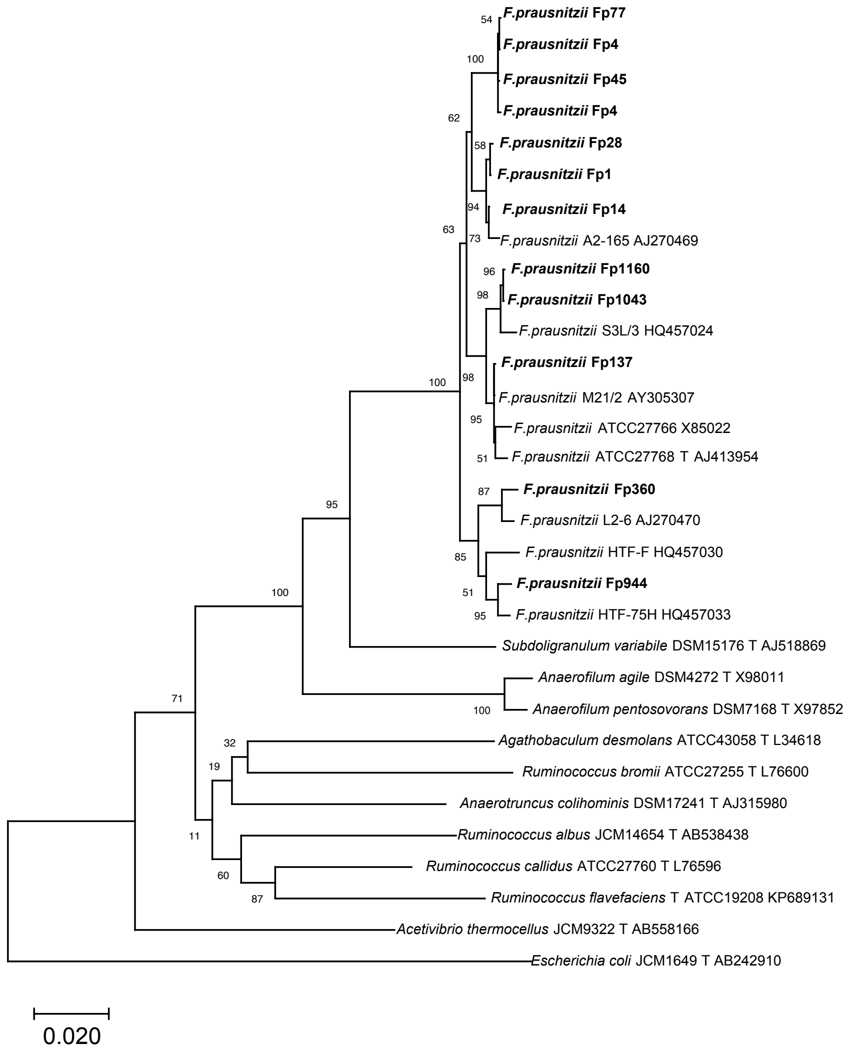

Figure S3. *F. prausnitzii* strains were isolated as candidates for gut microbiome-based intervention in MCI. Related to Figure 2.

Phylogenetic tree showing the relationships of the 12 *F. prausnitzii* isolates with other members of *Ruminococcaceae* based on 16S rRNA gene sequences. A neighbor-joining phylogenetic tree was constructed using MEGA. Bootstrap values (expressed as a percentage of 1,000 replications) are shown at branching points. The scale bar indicates the number of substitutions per nucleotide position. *F. prausnitzii* isolates in this study are highlighted in bold. Sequence accession numbers are shown at the last part of the strain name. “T” is the abbreviation for “type strain”.

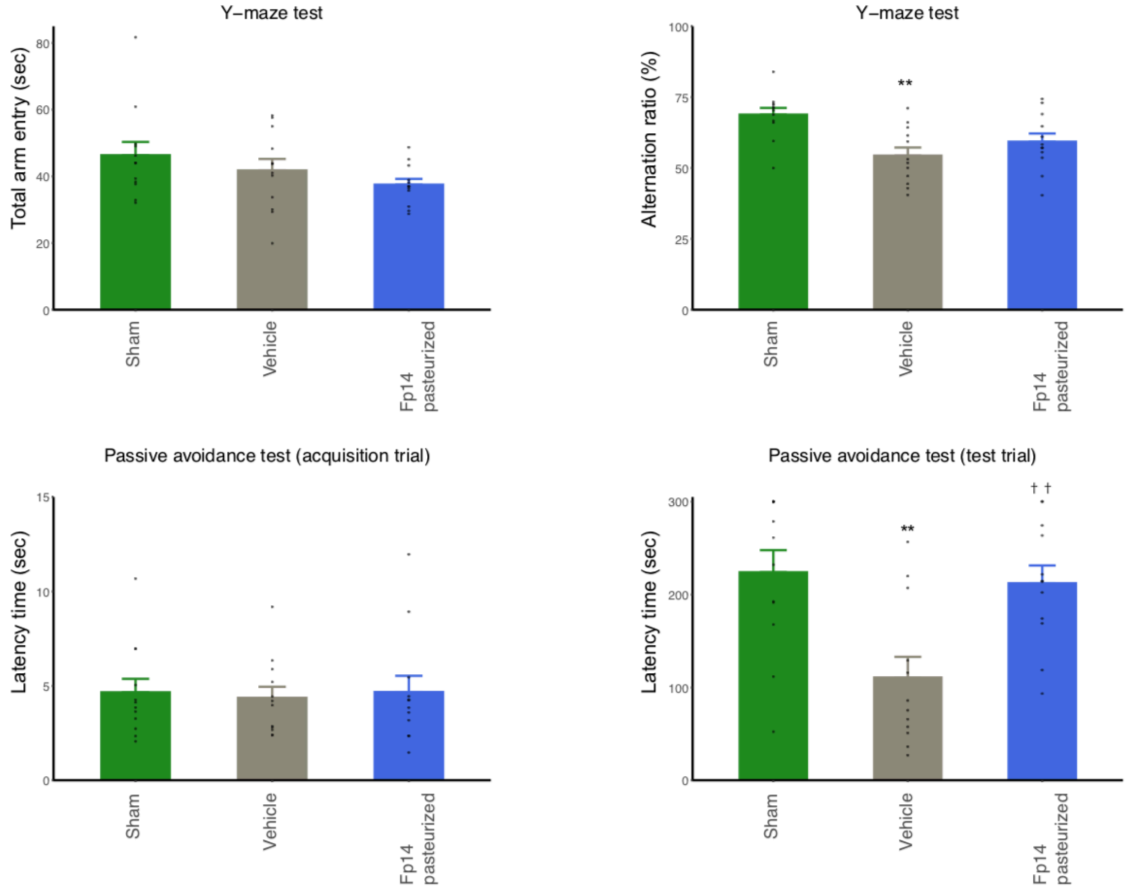

Figure S4. Pasteurized Fp14 reproducibly improved Aβ-induced cognitive impairment. Related to Figure 4.

Pasteurized Fp14 were orally administered to mice injected i.c.v. with Aβ25-35. Cognitive performance was evaluated by the Y-maze test and the passive avoidance test. (A) Bar plot showing the total entry time in the Y-maze test. (B) Bar plot showing the alternation ratio in the Y-maze test. (C) Bar plot showing the latency time of the acquisition trial in the passive avoidance test. (D) Bar plot showing the latency time of the test trial in the passive avoidance test. All values are expressed as the mean + S.E. (n=12, biological replicates). $^{**}p < 0.01$ by two-sided unpaired Student's *t*-test (sham-operation vs vehicle); $^{\dagger\dagger}p < 0.01$ by two-sided unpaired Student's *t*-test (vehicle vs Fp14 pasteurized).

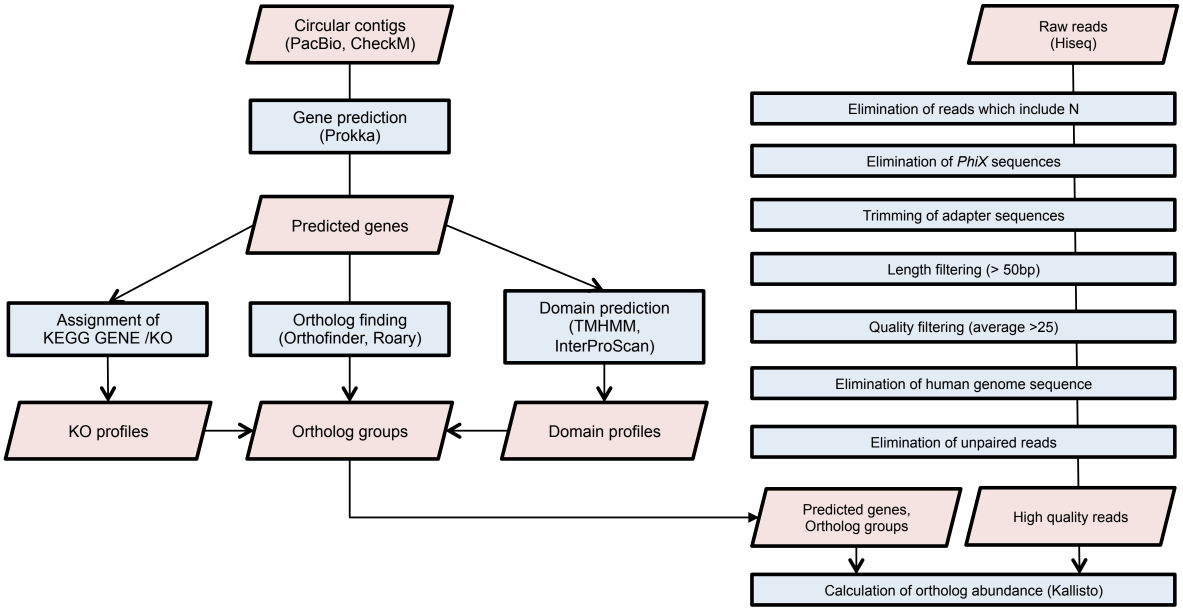

Figure S5. Pipeline for whole-genome comparison and whole-metagenome shotgun sequencing analysis. Related to Figure 5 and 6.

Regarding the whole-genome comparison, circular contigs from Pacbio underwent a quality control check by CheckM and gene prediction by Prokka. Protein domains were predicted using InterProScan, and TMHMM. Genes were annotated with KEGG genes and KEGG Orthologs (KOs) using DIAMOND. Orthologs were identified by using two different ortholog finding tools, Roary and Orthofinder, to enhance the precision of the analysis. KOs and domain profiles were assigned to each ortholog. Regarding the whole-metagenome shotgun sequencing analysis, raw reads first underwent a quality control to generate high-quality reads. The abundance of each gene from the isolated strains was evaluated by Kallisto. Each gene was categorized into an ortholog group, which was identified by Roary or Orthofinder, and the abundance of the orthologs was calculated (see also Methods Details).

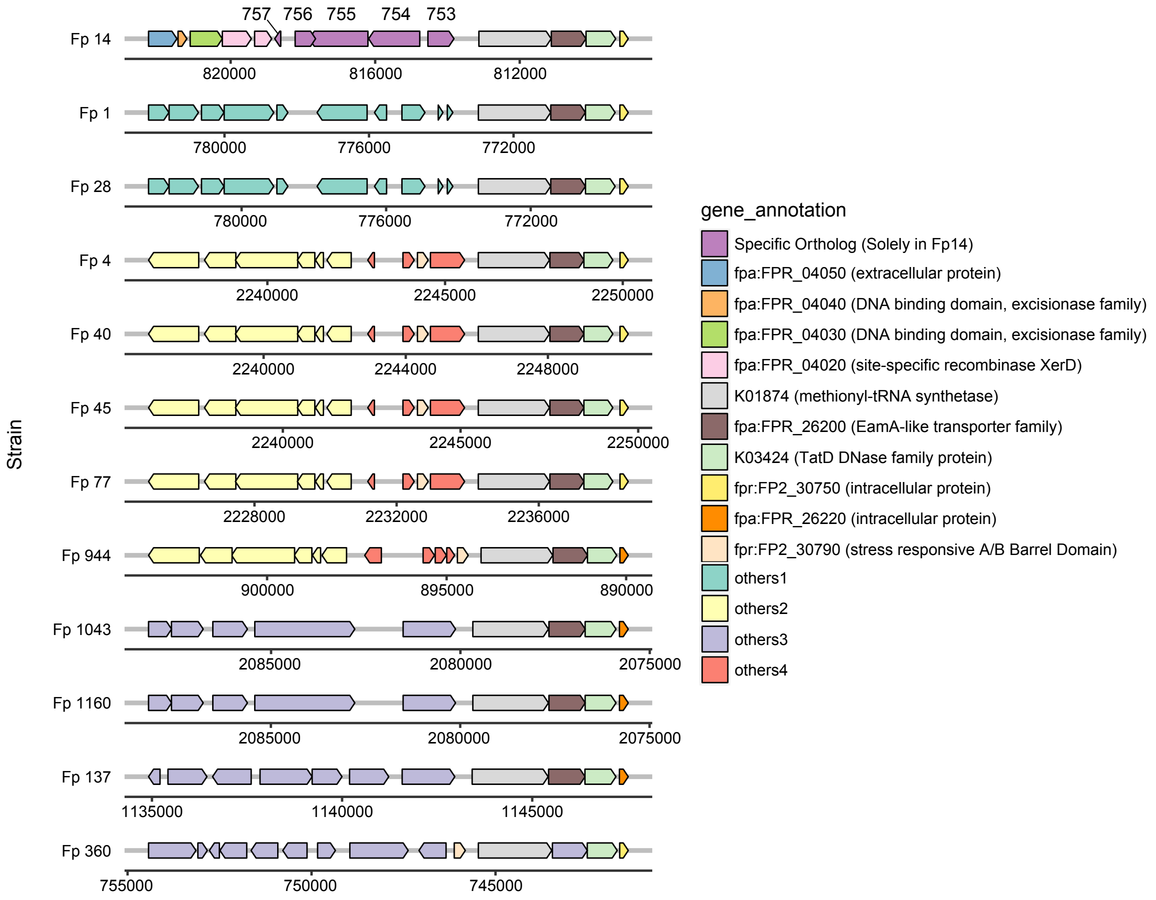

Figure S6. Whole-genome comparison revealed specific orthologs in the Fp14. Related to Figure 5.

Complete genomes of the 12 *F. prausnitzii* isolates were obtained using a PacBio sequencer. Specific orthologs were identified by using different ortholog finding tools, Roary and Orthofinder. Orthologs found only in specific strains or orthologs that contained KEGG Orthologs (KOs) found only in specific strains were defined as “specific orthologs”. KOs and domain profiles were assigned to each ortholog using DIAMOND, InterProScan, and TMHMM. 5 of 150 specific orthologs found only in Fp14 are shown.

Table S1. The characteristics of the selected subjects. Related to Figure 2 and 6.

Table showing the characteristics of the selected subjects in this study. All values except gender are expressed as the mean ± S.D. *P* value except for gender was calculated by Wilcoxon rank sum test (vs the healthy group). *P* value for gender was calculated by Fisher's exact test (vs the healthy group).

Number	Healthy (n = 21)	MCI (n = 15)	AD (n = 7)	*p* value (HvsM)	*p* value (HvsA)
Age (years)	72.7±5.3	79.8±6.1	83.0±9.3	0.0014	0.0092
Gender (n, % Female)	13, 61.9	9, 60.0	5, 71.4	1	1
BMI (kg/m^2)	22.7±3.7	24.4±2.3	22.9±2.7	0.1828	0.8735
MMSE (score)	29.5±0.7	26.9±2.3	15.6±4.3	0.0001	<0.0001
MoCA-J (score)	27.6±1.5	19.8±2.7	8.4±4.0	<0.0001	<0.0001
Education (years)	11.2±1.7	9.8±3.6	8.3±2.0	0.2146	0.0025

Table S2. The completeness and the contamination of the genomes of the 12 isolated *F. prausnitzii*. Related to Figure 5.

All genomes were estimated to be ≥95% complete with ≤5% contamination by CheckM, making them excellent data sets for whole-genome comparison(Parks et al., 2015).

Strain	Completeness (%)	Contamination (%)	Strain heterogeneity (%)
Fp1	100	0	0
Fp4	100	0	0
Fp14	100	0	0
Fp28	100	0	0
Fp40	100	0	0
Fp45	100	0	0
Fp77	100	0	0
Fp137	100	0	0
Fp360	100	0	0
Fp944	100	0	0
Fp1043	100	0	0
Fp1160	100	0.34	100
Fp1233	100	0	0
