# Supplementary material for: Identification of Faecalibacterium prausnitzii strains for gut microbiome-based intervention in Alzheimer’s-type dementia
Source: Cell Rep Med. 2021 Sep 14;2(9):100398. doi: 10.1016/j.xcrm.2021.100398 (PMC8484692; doi:10.1016/j.xcrm.2021.100398)
Supplement: Document S2. Article plus supplemental information [file mmc8.pdf]

Cell Reports
Medicine

Article

Identification of *Faecalibacterium prausnitzii* strains for gut microbiome-based intervention in Alzheimer's-type dementia

Graphical abstract

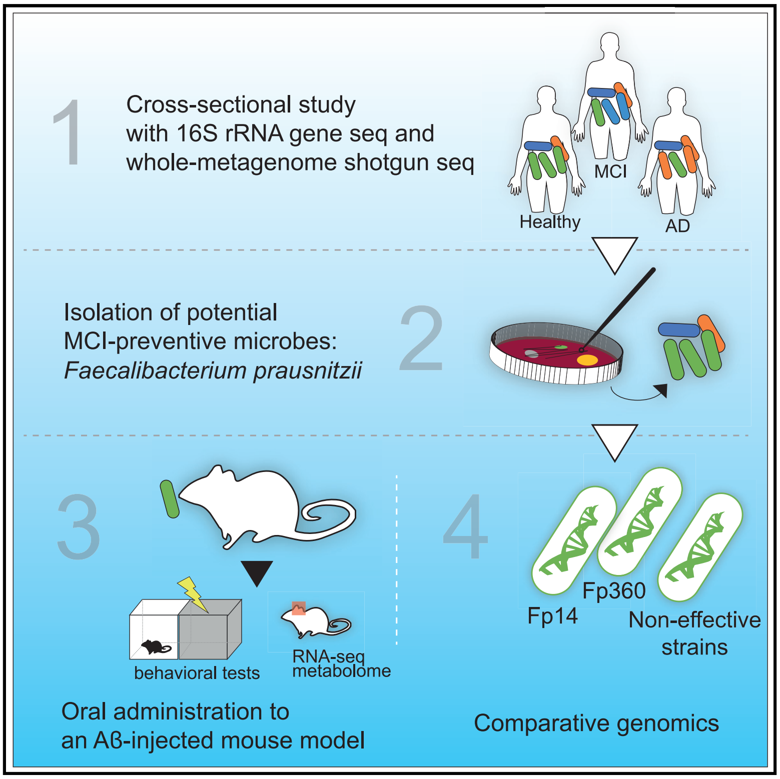

Highlights

- *F. prausnitzii* decreases in subjects with MCI and correlates with cognitive test scores
- Isolated *F. prausnitzii* strains improve Aβ-induced cognitive impairment in mice
- Comparative genomics reveals specific orthologs in the effective strains
- Effects of *F. prausnitzii* relate to oxidative stress and mitochondrial function

Authors

Atsushi Ueda, Shoji Shinkai, Hirotsugu Shiroma, ..., Kazunari Ushida, Akihiko Kitamura, Takuji Yamada

Correspondence

takuji@bio.titech.ac.jp

In brief

Ueda et al. observe a reduction of *F. prausnitzii* in volunteers with MCI and show that isolated *F. prausnitzii* strains from healthy volunteers improve cognitive impairment in mice. Using integrated whole-genome and metagenome analyses, they identify *F. prausnitzii* strains with specific orthologs as candidates for gut microbiome-based intervention in AD.

Ueda et al., 2021, Cell Reports Medicine *2*, 100398
September 21, 2021 © 2021 The Author(s).
https://doi.org/10.1016/j.xcrm.2021.100398

Cell Reports Medicine

CellPress
OPEN ACCESS

Article

Identification of *Faecalibacterium prausnitzii* strains for gut microbiome-based intervention in Alzheimer's-type dementia

Atsushi Ueda,[1,4] Shoji Shinkai,[2,5] Hirotsugu Shiroma,[1] Yu Taniguchi,[2,6] Sayaka Tsuchida,[3] Takahiro Kariya,[4] Tomohiro Kawahara,[4] Yodai Kobayashi,[4] Noriyuki Kohda,[4] Kazunari Ushida,[3] Akihiko Kitamura,[2] and Takuji Yamada[1,7,*]

[1]School of Life Science and Technology, Tokyo Institute of Technology, Tokyo, Japan
[2]Research Team for Social Participation and Community Health, Tokyo Metropolitan Institute of Gerontology, Tokyo, Japan
[3]College of Bioscience and Biotechnology, Chubu University, Aichi, Japan
[4]Otsu Nutraceuticals Research Institute, Otsuka Pharmaceutical Co., Ltd., Shiga, Japan
[5]Kagawa Nutrition University, Saitama, Japan
[6]Center for Health and Environmental Risk Research, National Institute for Environmental Studies, Ibaraki, Japan
[7]Lead contact
*Correspondence: takuji@bio.titech.ac.jp
https://doi.org/10.1016/j.xcrm.2021.100398

SUMMARY

Evidence linking the gut-brain axis to Alzheimer's disease (AD) is accumulating, but the characteristics of causally important microbes are poorly understood. We perform a fecal microbiome analysis in healthy subjects and those with mild cognitive impairment (MCI) and AD. We find that *Faecalibacterium prausnitzii* (*F. prausnitzii*) correlates with cognitive scores and decreases in the MCI group compared with the healthy group. Two isolated strains from the healthy group, live Fp360 and pasteurized Fp14, improve cognitive impairment in an AD mouse model. Whole-genome comparison of isolated strains reveals specific orthologs that are found only in the effective strains and are more abundant in the healthy group compared with the MCI group. Metabolome and RNA sequencing analyses of mouse brains provides mechanistic insights into the relationship between the efficacy of pasteurized Fp14, oxidative stress, and mitochondrial function. We conclude that *F. prausnitzii* strains with these specific orthologs are candidates for gut microbiome-based intervention in Alzheimer's-type dementia.

INTRODUCTION

Approximately 50 million people have dementia, and nearly 10 million new cases of dementia occur every year.[1] The elevated prevalence of Alzheimer's disease (AD), which accounts for approximately 60%–70% of total dementia, has become a fundamental health issue worldwide.[1] Various therapeutic approaches to AD have been examined but failed to show disease-modifying effects, presumably because of wrong timing of administration in the progression of AD.[2] Recent clinical studies show that pathological changes, which are characterized by β-amyloid (Aβ) plaques and neurofibrillary tangles of hyperphosphorylated tau, start 20 years before onset of clinical symptoms of AD,[3] and early intervention with a human monoclonal antibody that selectively targets aggregated Aβ, showed therapeutic potential.[4] These results indicate that early diagnosis and intervention would be necessary, and, in terms of prevention, intervention before or during mild cognitive impairment (MCI), the presymptomatic stage of AD, is regarded as promising.[5]

It is becoming increasingly recognized that the gut microbiota is one of the key regulators of gut-brain function.[6] The microbiota and the brain communicate with each other via various routes, including the immune system, the vagus nerve, and the enteric nervous system (microbiota-gut-brain-axis).[6] Psychobiotics (targeted microbiota interventions that support good brain health via microbiota-gut-brain-axis) are regarded as promising for development of disease interventions.[6] Evidence of the relationship between the gut microbiota and brain diseases such as autism, Parkinson's disease, multiple sclerosis, and even AD is accumulating.[7] For example, antibiotic-induced perturbations and germ-free intervention decrease Aβ plaque deposition in AD mice.[7] At the human level, several cross-sectional studies have reported a relationship between the gut microbiota and MCI or AD.[8,9] For example, one cross-sectional study identified that the prevalence of *Bacteroides* was lower in the control group than in the MCI group.[8] Another cross-sectional study showed a reduction in *Blautia* and *Ruminococcus* in the AD group compared with the healthy group.[9] Thus, we hypothesized that there would be a causal relationship between specific microbes and cognitive function in MCI, the prodromal stage of dementia, and that specific microbes would become promising candidates for gut microbiome-based preventive intervention in MCI.

© 2021 The Author(s).
This is an open access article under the CC BY license (http://creativecommons.org/licenses/by/4.0/).

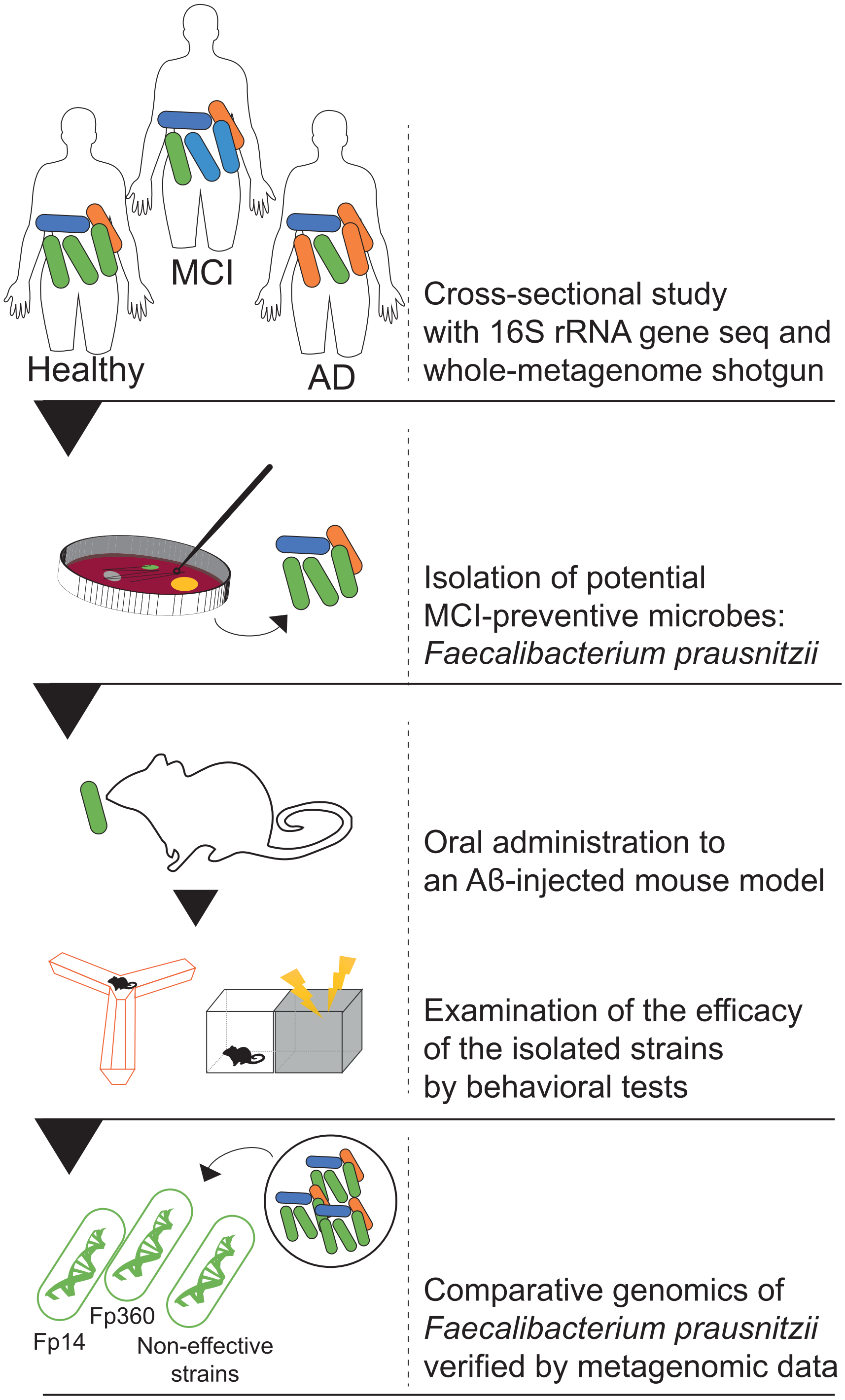

Figure 1. Schematic of this study
To find and isolate potential MCI-preventive microbes for gut microbiome-based intervention in MCI, a cross-sectional study was performed to compare gut microbial structures of healthy, MCI, and AD groups by fecal 16S rRNA gene sequencing. The comparative analysis of gut microbial composition led us to select and isolate *F. prausnitzii* as the most promising candidate for prevention of MCI. Part of the causal relationships between *F. prausnitzii* isolates and cognitive function was examined using an AD mouse model. The effective strains Fp14 and Fp360 were selected based on the animal study results, and whole-genome comparison of the effective strains with the non-effective strains identified specific orthologs that were found only in the effective strains. Metagenomics data from the same cross-sectional study verified that some of these specific orthologs were more abundant in the healthy group than in the MCI group, which implied a relationship of specific orthologs with MCI and, in part, the mechanism of the effect in different bacterial forms (pasteurized or live).

Therefore, in the present study, to find and isolate potential MCI-preventive microbes, we compared the gut microbial composition of healthy, MCI, and AD groups by fecal 16S rRNA gene sequencing (Figure 1). The comparative analysis of gut microbial composition guided us toward selection and isolation of *Faecalibacterium prausnitzii* (*F. prausnitzii*) as the most promising candidate for prevention of MCI. To examine part of the causal relationships between *F. prausnitzii* and cognitive function, we administered isolated *F. prausnitzii* to an AD mouse model and successfully identified two effective strains, Fp14 and Fp360. We used a validated Aβ-injected mouse model[10] that is used widely in the research area of AD.[11–16] This mouse model displays specific dysfunction of memory processes, and there are symptomatic and pathophysiological similarities of this model with human AD (Method details).[10] Therefore, this validated animal model could be useful for evaluating candidates for intervention in Alzheimer's-type dementia through behavioral experiments. After we determined Fp14 and Fp360 to be effective strains, whole-genome comparison and metagenome shotgun sequencing identified specific orthologs that were found only in these effective strains and were more abundant in the healthy group than in the MCI group. In addition, metabolome and RNA sequencing (RNA-seq) analysis exhibited a relationship between the efficacy of pasteurized Fp14, oxidative stress, and mitochondrial function in the brain. We conclude that *F. prausnitzii* strains with these specific orthologs are candidates for gut microbiome-based intervention in Alzheimer's-type dementia.

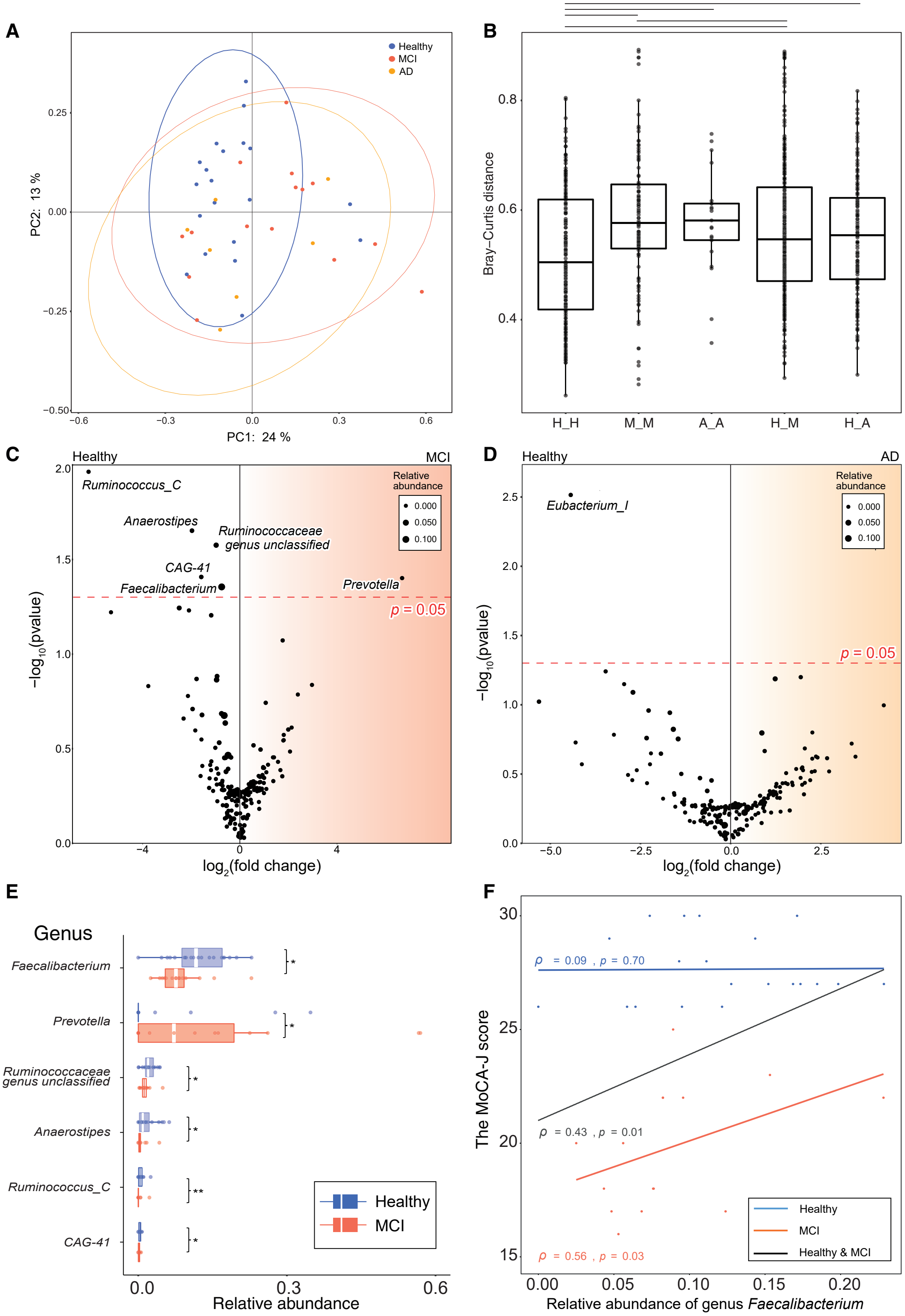

(legend on next page)

RESULTS

F. prausnitzii decreased in the MCI group compared with the healthy group and correlated with cognitive test scores

We first performed our own cross-sectional study to identify potential MCI-preventive microbes. Fecal samples of the healthy, MCI, and AD groups were collected in the town of Kusatsu in Gunma, Japan. The participant characteristics and a flow diagram of subject selection are shown in Table S1 and Figure S1. Gut microbial composition was analyzed by 16S rRNA gene sequencing. Taxonomic assignment at the genus level detected 129 genera. We examined the overall genus composition profiles by performing principal coordinates analysis (PCoA) and permutational multi-variate ANOVA (PERMANOVA) with Bray-Curtis distance (Figure 2A). Genus composition had a tendency to differ between the healthy and the MCI group ($R^2 = 0.0465$, $p = 0.0968$) but not between the healthy and the AD group ($R^2 = 0.0534$, $p = 0.1423$) (Figure 2A). The within-group Bray-Curtis distance was significantly higher in the MCI and AD groups than in the healthy group, which confirmed the PCoA trend ($p = 2.92 \times 10^{-5}$, $p = 0.0203$, respectively) (Figure 2B). The Shannon-Wiener alpha-diversity index did not differ between the groups (Figure S2). Among 129 genera, differential abundance analysis by ALDEx2 revealed that the abundances of 6 genera were significantly different between the healthy and the MCI group (Figure 2C). *Faecalibacterium*, *Ruminococcaceae* genus unclassified, *Anaerostipes*, *Ruminoccocus_C*, and *CAG-41* decreased significantly in the MCI group compared with the healthy group. *Prevotella* increased significantly in the MCI group compared with the healthy group (Figure 2C). The abundance of *Eubacterium_I* was significantly different between the healthy and the AD group (Figure 2D). The difference in relative abundances is also shown in Figures 2E and S2. In terms of AD prevention, intervention before or during MCI is regarded as promising.[5] We therefore considered that, for selection of candidate psychobiotics for AD prevention, a comparison of the healthy and the MCI group is more suitable so than a comparison of the healthy and the AD group. Thus, we focused on microbes that were not only correlated with Montreal Cognitive Assessment Japanese version (MoCA-J) scores but also abundant in the healthy group but decreased in the MCI group, and the correlation between the relative abundance of bacterial genera and the MoCA-J scores of the healthy and the MCI group was calculated. The abundances of 6 genera that showed significant differences between the healthy and the MCI group were correlated significantly with MoCA-J scores (Figure 2F; Table S4). Among them, *F. prausnitzii* was the most abundant and was decreased significantly in the MCI group compared with the healthy group (Figures 2C and 2E).

F. prausnitzii strains were isolated as candidates for gut microbiome-based intervention in MCI

In addition to its correlation with MoCA-J scores and its high abundance (Figures 2C, 2E, and 2F), *F. prausnitzii* has recently attracted considerable attention as a next-generation health-promoting bacterium.[17,18] Therefore, we selected *F. prausnitzii* as the most promising candidate for prevention of MCI and aimed to isolate these potential MCI-preventive microbes from the same subjects who underwent the gut microbial composition analysis. As a result, 10 *F. prausnitzii* strains were isolated among approximately 4,000 total bacterial isolates. In parallel, we performed an additional isolation experiment, and 2 *F. prausnitzii* strains were isolated from a healthy middle-aged volunteer; thus, in total, 12 *F. prausnitzii* strains were used for further analysis. The phylogenetic relationships of the 12 isolates with other *F. prausnitzii* strains and members of *Ruminococcaceae* are shown in Figure S3.

F. prausnitzii isolates improved Aβ-induced cognitive impairment

To examine part of the causal relationship between *F. prausnitzii* and cognitive function, 12 isolates of *F. prausnitzii* were administered orally (p.o.) to mice that were injected Aβ25-35 intracerebroventricularly (i.c.v.). The vehicle group (i.c.v., Aβ; p.o., saline) showed a significant reduction in alternation behavior in the Y-maze test compared with the sham operation group (i.c.v., distilled water; p.o., saline), suggesting that mice injected with Aβ25-35 showed impaired working memory (Figure 3B). However, among the 12 isolates, oral administration of live Fp14 ($p = 0.0070$), Fp28 ($p = 0.0181$), Fp77 ($p = 0.0323$), and Fp360 ($p = 0.0001$) (i.c.v., Aβ; p.o., either of the live bacteria suspended in culture medium) improved this alternation behavior impairment compared with medium-administered mice (i.c.v., Aβ; p.o., culture medium) in the Y-maze test (Figure 3B). There were no significant difference in the total number of entries into the three arms among the groups except for the donepezil group and Fp4, suggesting that Fp14, Fp28, Fp77, and Fp360 did not affect locomotor activity (Figure 3A). Regarding the passive avoidance (PA) test,

Figure 2. *F. prausnitzii* decreased in the MCI group compared with the healthy group and correlated with cognitive test scores

(A) Scatterplot showing the result of principal coordinates analysis (PCoA) with Bray-Curtis distance.

(B) Boxplot showing the interquartile range (IQR) of the within-group Bray-Curtis distance, confirming the PCoA trend. The black horizontal lines show $p < 0.05$, which was analyzed by Wilcoxon rank-sum test (H, Healthy; M, MCI; A, AD).

(C and D) Volcano plot showing genera whose abundance was significantly different (C) between the healthy ($n = 20$) and the MCI group ($n = 15$) or (D) between the healthy and the AD group ($n = 7$) by differential abundance analysis (ALDEx2). The x axis shows the $\log_2$ fold change of (C) the MCI or (D) the AD group over the healthy group. The y axis shows the $-\log_{10}$ of the p value, analyzed by Wilcoxon rank-sum test. The red horizontal line indicates $p = 0.05$. The size of the circle shows the median abundance of each genus. The abundance of species *F. prausnitzii* and genus *Faecalibacterium* are the same because *F. prausnitzii* is the sole species belonging to the genus *Faecalibacterium*.

(E) Boxplot showing the IQR of the relative abundance of the genera between the healthy and MCI group. Six genera whose abundance was significantly different between the healthy and the MCI group are shown (Wilcoxon rank-sum test, $^{**}p < 0.01$, $^{*}p < 0.05$).

(F) Scatterplot showing Spearman's ρ and p value between the relative abundance of *F. prausnitzii* and the Montreal Cognitive Assessment Japanese version (MoCA-J) score of the healthy group (blue), the MCI group (red), and the healthy and the MCI groups (black).

See also Figures S1–S3 and Tables S1, S3, and S4.

CellPress
OPEN ACCESS

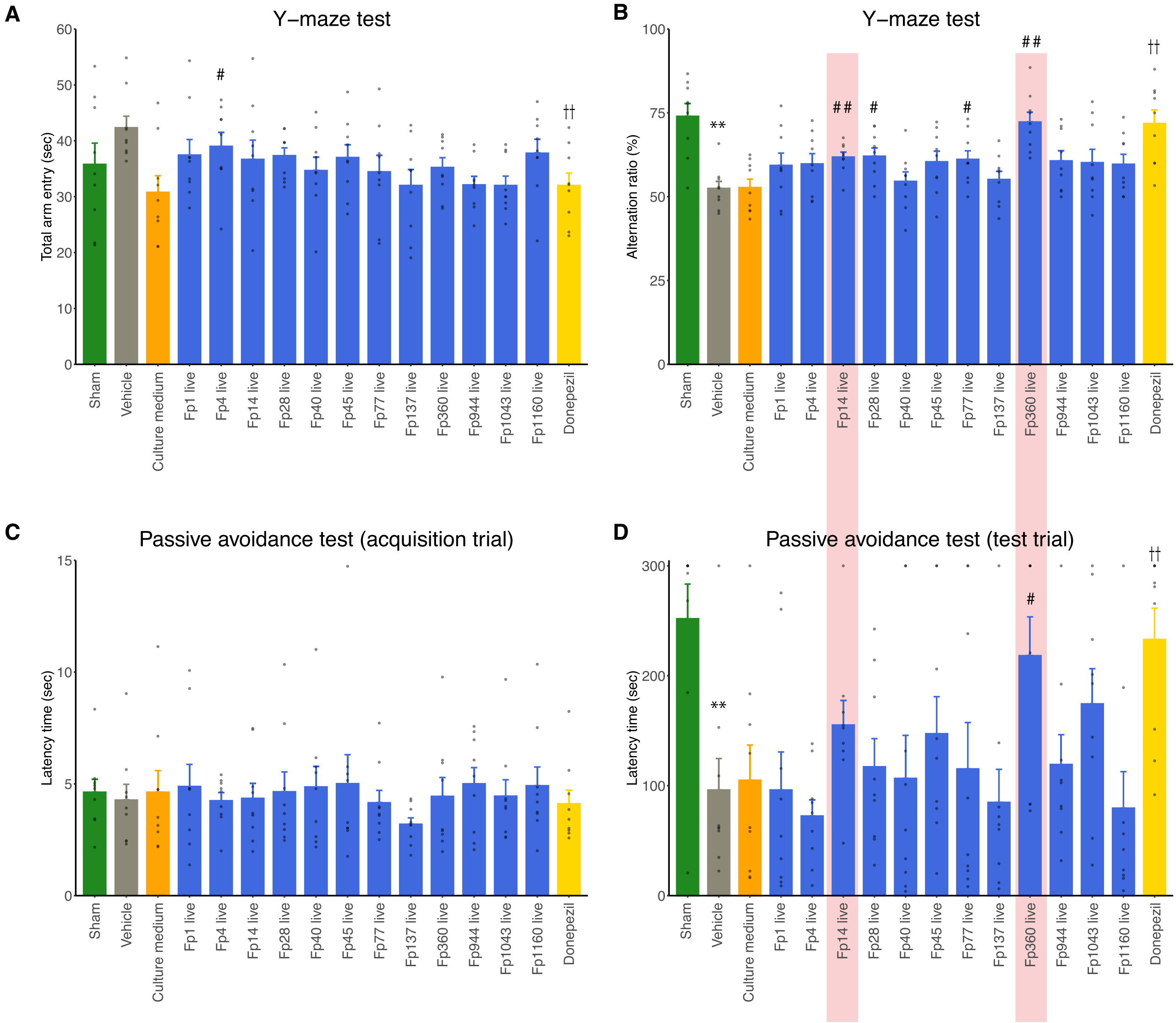

Figure 3. *F. prausnitzii* isolates improved Aβ-induced cognitive impairment

12 live isolates of *F. prausnitzii* were administered orally to mice that were injected i.c.v. with Aβ25-35. Cognitive performance was evaluated by Y-maze test and PA test.

(A) Bar plot showing total entry time in the Y-maze test.

(B) Bar plot showing the alternation ratio in the Y-maze test.

(C) Bar plot showing the latency time of the acquisition trial in the PA test.

(D) Bar plot showing the latency time of the test trial in the PA test.

All values are expressed as the mean + SE (n = 9, biological replicates). $^{**}p < 0.01$ by two-sided unpaired Student's t test (sham operation versus vehicle); $^{\#}p < 0.05$, $^{\#\#}p < 0.01$ by two-sided unpaired Student's t test (versus culture medium); $^{\dagger\dagger}p < 0.01$ by two-sided unpaired Student's t test (versus vehicle).

the vehicle group had a significantly lower latency time than the sham operation group during the test trial, but this was improved by oral administration of live Fp360 ($p = 0.0303$) (Figure 3D), indicating that live Fp360 could attenuate the long-term memory dysfunction of Aβ-injected mice. There was no significant difference in the latency time among groups in the acquisition trial (Figure 3C). Considering the results of these two tests, we focused on Fp14 and Fp360 for further analysis as the most promising candidates among the 12 strains of *F. prausnitzii* because they showed the third-highest and highest potential, respectively, in the Y-maze test and PA test.

The selected Fp14 and Fp360 improved Aβ-induced cognitive impairment in different bacterial forms (pasteurized or live)

To investigate whether efficacy was derived from the selected *F. prausnitzii* itself or its metabolites, live Fp14 or Fp360, pasteurized Fp14 or Fp360, and their corresponding culture supernatant

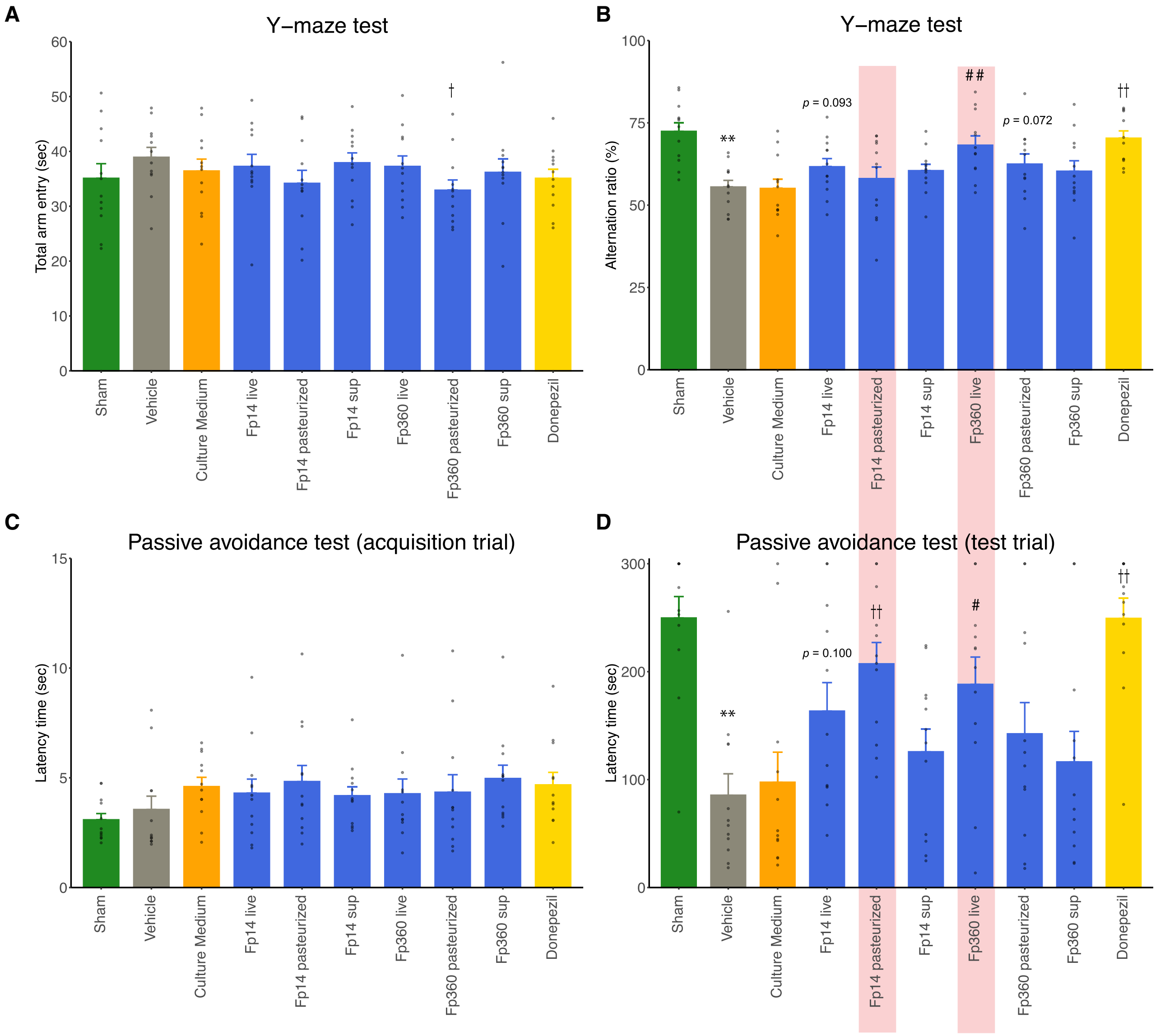

Figure 4. The selected Fp14 and Fp360 improved Aβ-induced cognitive impairment in different bacterial forms (pasteurized or live)
Live Fp14 or Fp360, pasteurized Fp14 or Fp360, and their corresponding culture supernatant were administered orally to mice that were injected i.c.v. with Aβ25-35. Cognitive performance was evaluated by Y-maze test and PA test.
(A) Bar plot showing total entry time in the Y-maze test.
(B) Bar plot showing the alternation ratio in the Y-maze test.
(C) Bar plot showing the latency time of the acquisition trial in the PA test.
(D) Bar plot showing the latency time of the test trial in the PA test.
All values are expressed as the mean + SE (n = 12, biological replicates). **p < 0.01 by two-sided unpaired Student's t test (sham operation versus vehicle); #p < 0.05, ##p < 0.01 by two-sided unpaired Student's t test (versus culture medium); †p < 0.05, ††p < 0.01 by two-sided unpaired Student's t test (versus vehicle). See also Figure S4.

were administered orally to mice injected i.c.v. with Aβ25-35. Live Fp360 reproducibly and significantly improved Aβ-induced cognitive impairment in the Y-maze test (p = 0.0032) (Figure 4B) and the PA test (p = 0.0249) (Figure 4D). However, pasteurized Fp360 and its culture supernatant did not show significant improvement of Aβ-induced cognitive impairment (p = 0.0717, 0.2319 for the Y-maze test [Figure 4B]; p = 0.1197, 0.6402 for the PA test [Figure 4D]). Regarding Fp14, live Fp14 had a tendency to improve Aβ-induced cognitive impairment only in the Y-maze test (p = 0.0932; Figure 4B), with no effects on the PA test (p = 0.1002; Figure 4D) and, thus, did not show any reproducible significant improvement in the Y-maze test compared with the results of the previous animal study (Figure 3B). However, to our surprise, pasteurized Fp14 improved Aβ-induced cognitive impairment in the PA test (p = 0.0003; Figure 4D), and this efficacy was shown to be reproducible (p = 0.0023;

CellPress
OPEN ACCESS

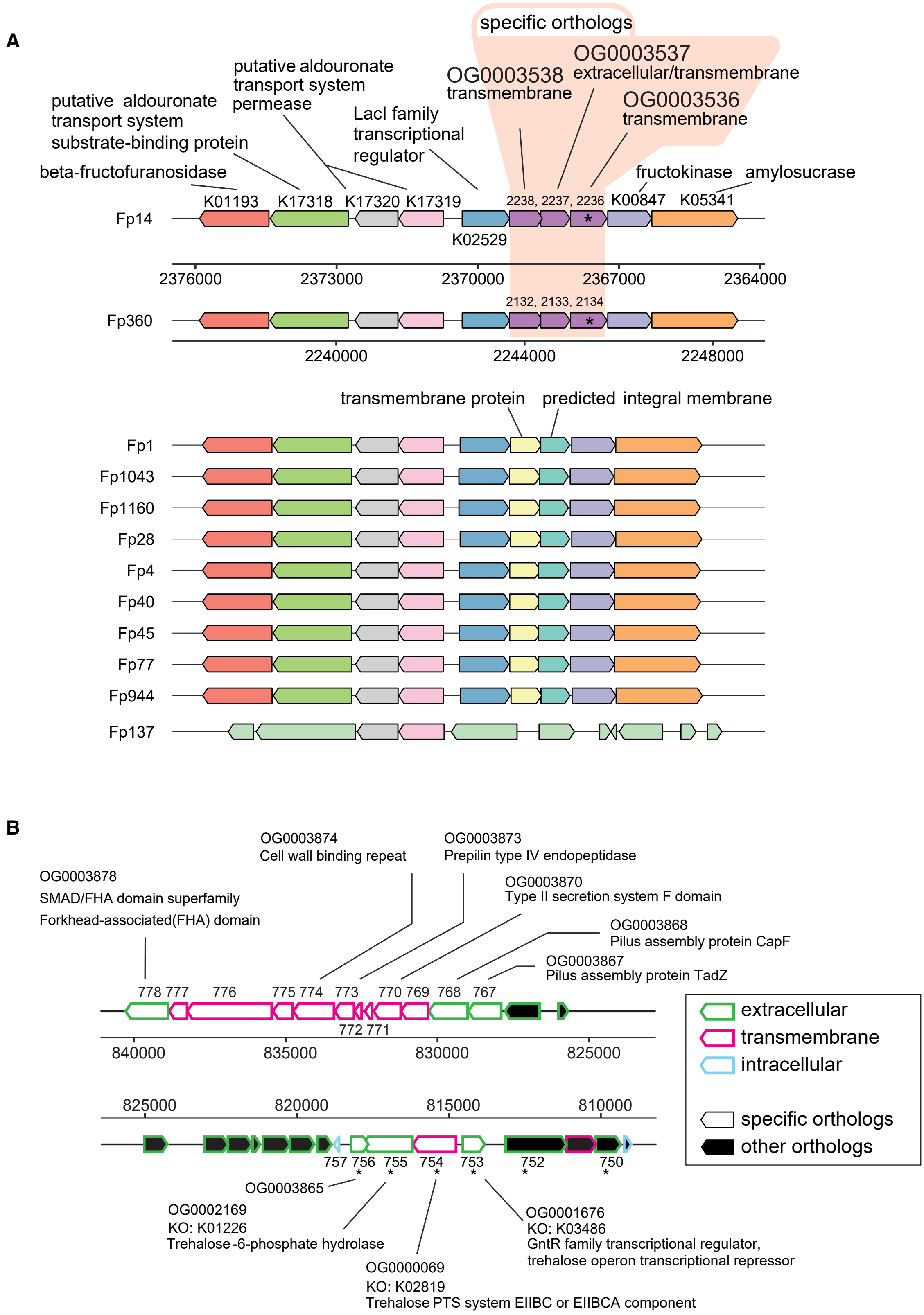

(legend on next page)

Figure S4). On the other hand, the culture supernatant of Fp14 did not significantly reduce Aβ-induced cognitive impairment (p = 0.1263 for the Y-maze test [Figure 4B]; p = 0.4282 for the PA test, [Figure 4D]).

Whole-genome comparison revealed specific orthologs in the effective *F. prausnitzii* strains

We next hypothesized that Fp14 and Fp360 would share specific genomic features because of their shared efficacy in the PA test. Furthermore, we examined whether Fp14 and Fp360 each had their own specific genomic features because of the difference in their effective form (pasteurized for Fp14 or live for Fp360). Complete genomes of the 12 *F. prausnitzii* isolates were obtained, and specific orthologs were identified using a whole-genome comparison analytical pipeline (Figure S5). Orthologs found only in specific strains or orthologs that contained KEGG orthologs (KOs) found only in specific strains were defined as "specific orthologs."

Four specific orthologs were determined to be shared only by Fp14 and Fp360 when we compared their genome with those of the 10 other isolates (Table S5). Three of these 4 specific orthologs were adjacent to each other (Figure 5A).

Next, 150 specific orthologs were identified solely in Fp14 when we compared its genome with those of the other 11 isolates (Table S5). Five specific orthologs from genes 753–757 were adjacent to each other, and 3 of these 5 specific orthologs were related to trehalose metabolism (Figures 5B and S6; Table S5). Twelve specific orthologs from genes 767–778 were adjacent to each other, and 2 of these 12 specific orthologs were related to pilus assembly (Figure 5B; Table S5).

Furthermore, 214 specific orthologs were identified solely in Fp360 when we compared its genome with those of the other 11 isolates (Table S5). Some of these specific orthologs were related to cell division (Table S5).

Metagenome shotgun sequencing verified abundant orthologs in the healthy group

Furthermore, we sought to verify whether these specific orthologs have a connection to MCI and the ameliorating efficacy of these effective strains by using metagenomic data from the same cross-sectional study (Figure 2; Table S1). The abundance of specific orthologs in the metagenome was calculated and compared between the healthy and the MCI group. In addition, to investigate the functions of these specific orthologs, KOs and domain profiles were assigned to each ortholog (Figure S5).

Among the 4 specific orthologs that were shared only by Fp14 and Fp360, the abundance of one ortholog (OG0003536; Figure 5A) was significantly higher in the healthy group than in the MCI group (p = 0.0226; Figures 6A and 6B; Table S6), and this ortholog was predicted as a transmembrane protein by TMHMM (Figure 5A; Table S5).

Among the 150 specific orthologs that were found solely in Fp14, 4 specific orthologs (OG0003865, OG0002169, OG0000069, and OG0001676; Figure 5B) were significantly higher in the healthy group than in the MCI group (p = 0.0099, 0.0009, 0.0121, and 0.0024, respectively) (Figures 6A and 6B; Table S6). These 4 specific orthologs were adjacent to each other (Figure 5B) and predicted to be an extracellular protein or a transmembrane protein (Figure 5B; Table S5). Additionally, 3 of these 4 specific orthologs (OG0002169, OG0000069, and OG0001676) were related to trehalose metabolism (Figure 5B; Table S5).

Among the 214 specific orthologs that were found solely in Fp360, 3 specific orthologs (OG0004181, OG0000713, and OG0000772) were significantly higher in the healthy group than in the MCI group (p = 0.0416, 0.0162, and 0.0195, respectively) (Figures 6A and 6B; Table S6) and predicted to be extracellular proteins, transmembrane proteins, and intracellular proteins, respectively (Table S5). Additionally, 2 of 3 specific orthologs (OG0000713 and OG0000772) were related to cell division (Figure 6B; Table S5).

Metabolome and RNA-seq analysis explored the potential mechanism of action of pasteurized Fp14 in the brain

A limitation of using *F. prausnitzii* is its high sensitivity to oxygen, so the efficacy of Fp14 gained by pasteurization would be a feasible solution for use of *F. prausnitzii* as a preventive intervention material. Thus, we focused on pasteurized Fp14 for further analysis. We performed metabolome and RNA-seq analyses of the hippocampus gained from the behavioral animal experiments to explore the potential mechanism of efficacy. Metabolome analysis detected 355 annotated metabolites (Figure 7A; Table S7). Among them, pasteurized Fp14 significantly reduced thymine (p = 0.032; Figure 7B) and N^6-methyl-2′-deoxyadenosine (m6dA or 6mA, p = 0.033; Figure 7B) and had a tendency to reduce suberic acid (p = 0.072; Figure 7B). These metabolites have been reported to be related to oxidative stress and mitochondrial functions. RNA-seq analysis also detected 15 differentially expressed transcripts (Table S8). Among them, pasteurized Fp14 significantly reduced the transcripts of phosphofurin acidic cluster sorting protein 2 (PACS-2) (q = 3.29E−16 and q = 1.56E−16, respectively; Table S8), which has also been reported to be related to mitochondrial function.

Figure 5. Whole-genome comparison revealed specific orthologs in the effective *F. prausnitzii* strains

Complete genomes of the 12 *F. prausnitzii* isolates were obtained using a PacBio sequencer. Specific orthologs were identified by using different ortholog finding tools: Roary and Orthofinder. Orthologs found only in specific strains or orthologs that contained KEGG orthologs (KOs) found only in specific strains were defined as "specific orthologs."

(A) 3 of the 4 specific orthologs shared only by Fp14 and Fp360 were adjacent to each other. Specific orthologs shared only by Fp14 and Fp360 were not present in the other 10 isolated strains.

(B) 17 of 150 specific orthologs found solely in Fp14 were adjacent to each other.

Intergroup differences in ortholog abundance between the healthy and the MCI group were analyzed by Wilcoxon rank-sum test (*p < 0.05). Specific orthologs whose abundance was significantly higher in the healthy group than in the MCI group are indicated by asterisks. KOs and domain profiles were assigned to each ortholog using DIAMOND, InterProScan, and TMHMM. See also Figures S5 and S6 and Tables S2 and S5.

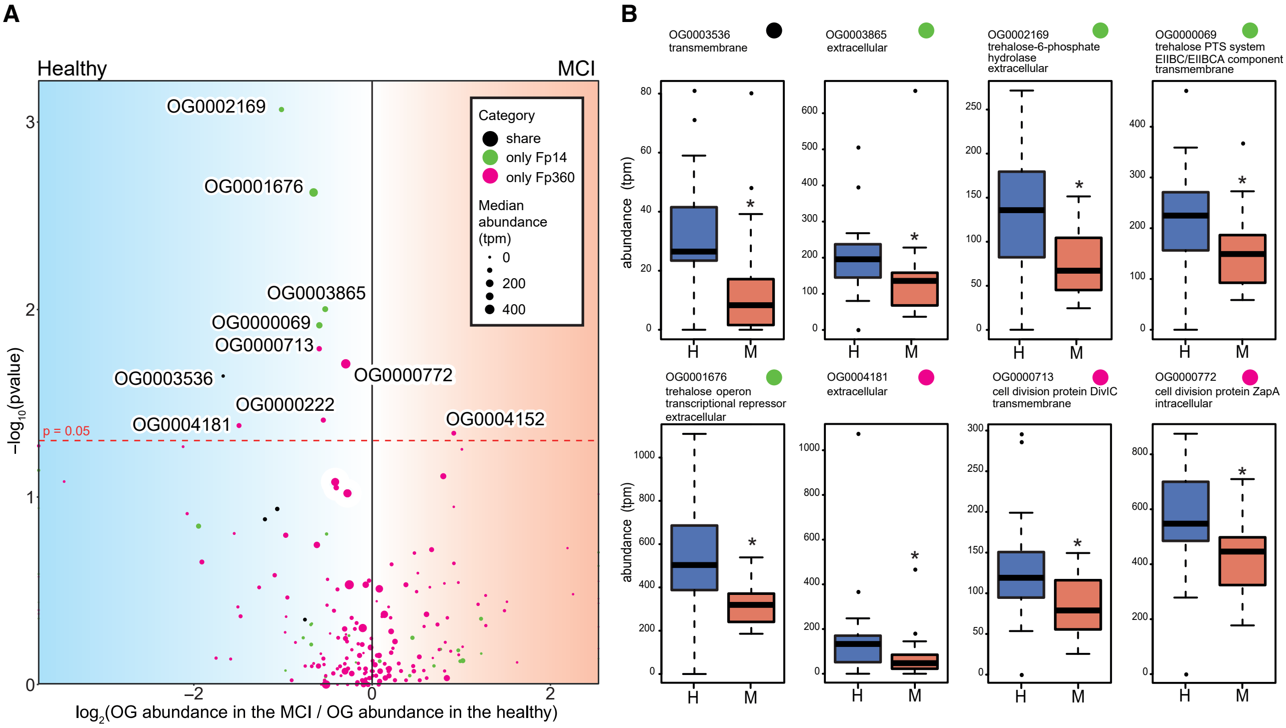

Figure 6. Metagenome shotgun sequencing verified abundant orthologs in the healthy group

(A) Volcano plot showing the specific orthologs whose abundance was significantly different between the healthy and the MCI group (Wilcoxon rank-sum test). Orthologs found only in specific strains or orthologs that contained KOs found only in specific strains were defined as "specific orthologs." The x axis shows the $\log_2$ of the median abundance in the MCI group over the median abundance in the healthy group. The y axis shows the $-\log_{10}$ of the p value analyzed by the Wilcoxon rank-sum test. The red horizontal line indicates $p = 0.05$. The size of the circles shows the median abundance of each ortholog. Colored circles show the category (black, shared only by Fp14 and Fp360; green, only Fp14; magenta, only Fp360).

(B) Boxplots showing the IQR of the abundance of the specific orthologs. Eight specific orthologs whose abundance was significantly higher in the healthy group than in the MCI group are shown (Wilcoxon rank-sum test, $^*p < 0.05$). Colored circles show the category (black, shared only by Fp14 and Fp360; green, only Fp14; magenta, only Fp360). These significant results were obtained when we used specific orthologs categorized by Orthofinder, and the same significant results were also observed when we used specific orthologs categorized by Roary (Table S6), except OG0000222. OG0000222 categorized by Orthofinder was significantly higher in the healthy group, but the ortholog that was categorized by Roary, which contained the same genes of Fp360 in OG0000222, was not significantly higher in the healthy group.

See also Figure S5 and Tables S1, S3, S5, and S6.

DISCUSSION

In the present study, we highlighted the characteristics of *F. prausnitzii* strains with specific orthologs as candidates for gut microbiome-based intervention in Alzheimer's-type dementia. The comparative analysis of gut microbial composition in the cross-sectional study guided us to select and isolate *F. prausnitzii*, and administration of the isolated *F. prausnitzii* strains to an AD mouse model demonstrated part of the causal relationship between strains of *F. prausnitzii* and cognitive function in MCI. Furthermore, whole-genome comparison and metagenome shotgun sequencing revealed specific orthologs that were found only in the effective strains and were more abundant in the healthy group than in the MCI group. In addition, metabolome and RNA-seq analysis exhibited the relationship between the efficacy of pasteurized Fp14, oxidative stress, and mitochondrial function in the brain.

In terms of AD prevention, intervention before or during MCI is regarded as promising.[5] We therefore considered that, for selection of candidate psychobiotics for AD prevention, a comparison of the healthy and the MCI group is more suitable than a comparison of the healthy and the AD group. *F. prausnitzii* decreased in the MCI group and had a positive correlation with cognitive test (MoCA-J) score (Figures 2C and 2F), and administration of either of the two of *F. prausnitzii* strains (pasteurized Fp14 and live Fp360) isolated from the subjects who participated in our cross-sectional study were shown to improve cognitive impairment in an AD mouse model (Figures 3 and 4). There were disparities in group size, gender, and age, as shown in Table S1, and there was a significant difference between the average age of the healthy and the MCI group. It is known that microbiome composition changes with age,[19] but there was no significant correlation between age and the abundance of *F. prausnitzii* in each group (Figure S2D), from which we speculate that the reduction of *F. prausnitzii* between the healthy and MCI group is related to the group difference. The results of our cross-sectional study between the healthy and the MCI group showed some differences from or similarities to the accumulated

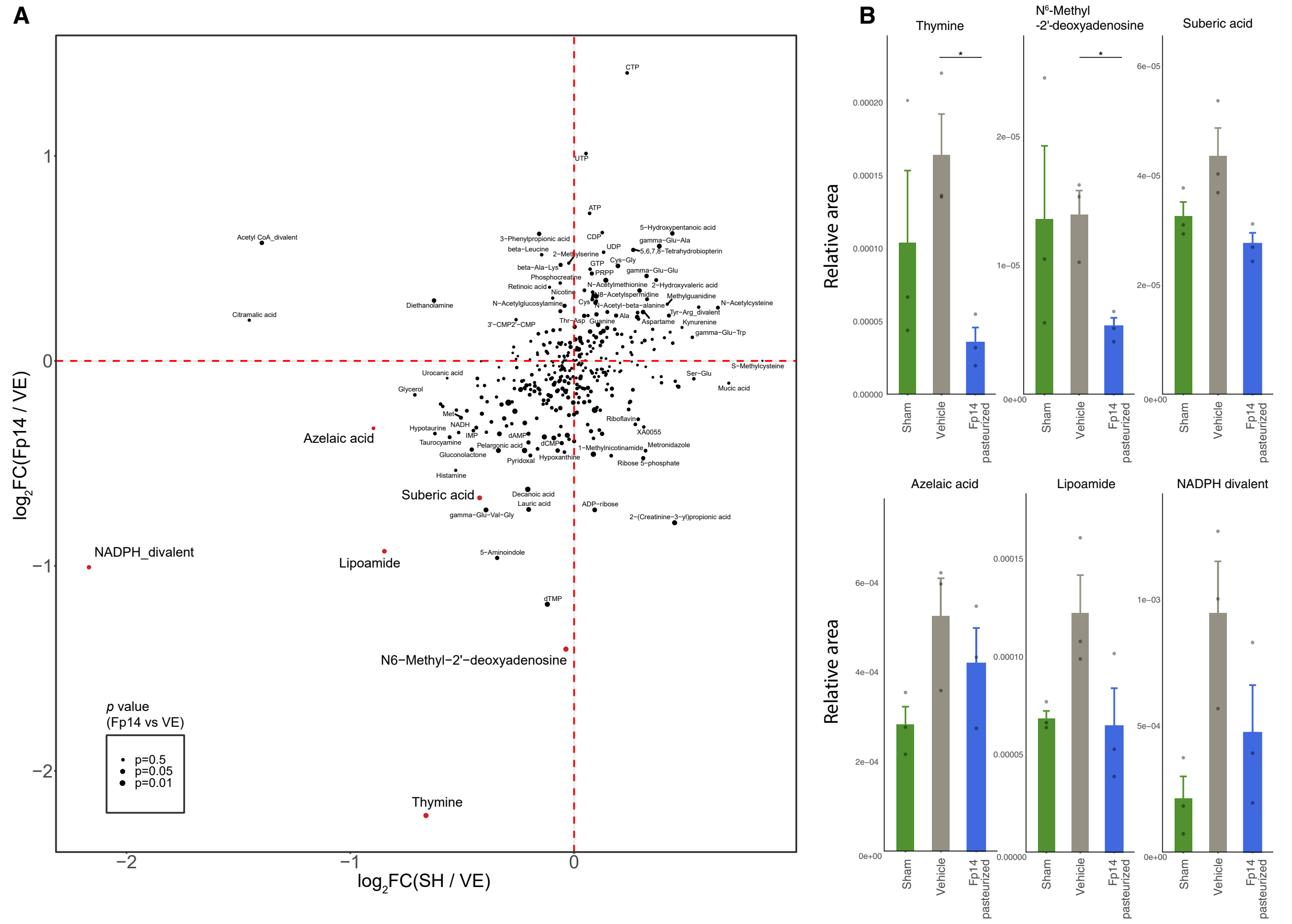

Figure 7. Metabolome analysis explored the potential mechanism of action of pasteurized Fp14 in the brain

(A) Scatterplot showing the result of the metabolome analysis. The x axis shows the log_2 fold change of the sham operation group over the vehicle group. The y axis shows log_2 fold change of the pasteurized Fp14-administered group over the vehicle group. The size of the circle shows the p value (two-sided unpaired Welch's t test) between the pasteurized Fp14-administered group and the vehicle group.

(B) Bar plot showing the relative areas of metabolites related to oxidative stress and mitochondrial function.

All values are expressed as the mean + SE (n = 3, biological replicates). *$p < 0.05$ by two-sided unpaired Welch's t test (versus vehicle). See also Table S7 and S8.

evidence of which we are aware.[8,20,21] One Japanese cross-sectional study reported that the prevalence of *Bacteroides* was lower in the control group than in the MCI group, but the number of *F. prausnitzii* was not referred to.[8] We speculate that the different gut microbial composition analysis method (our 16S rRNA gene sequencing and their terminal restriction fragment length polymorphism [T-RFLP] method) caused the different results, as often observed in other studies.[22,23] A Chinese cross-sectional study showed a reduction in *Faecalibacterium* in the MCI group compared with the healthy group, in accordance with our results,[20] but an American cross-sectional study did not.[21] We speculate that these differences or similarities come from differences in the subjects' backgrounds, such as geography, culture, and dietary habits, as discussed in another article.[24] Regarding our results for the healthy and the AD group, no reduction of *Faecalibacterium* and no increase of *Prevotella* were observed (Figures S2B and S2E), which was different from the comparison of the healthy and MCI group in our study. The reason for this is currently unknown, but this result was consistent with one American cohort.[25] However, a Chinese cohort and another American cohort showed a significant reduction of *Faecalibacterium* and a significant increase of *Prevotella denticola* in the AD group,[26,27] so we also speculate that these differences come from differences in the subjects' backgrounds.[24] Further research in multi-center cohorts is needed to elucidate whether the difference in *Faecalibacterium* is a universal phenomenon. Given the difficult interpretation of the long-term changes in lifestyle, including residence, diet, and disease progress, in AD[7] and for preventive intervention in the future, we focused on *F. prausnitzii*, which was not only correlated with cognitive test scores but also abundant in the healthy group and decreased in the MCI group (Figures 2C and 2F). Moreover, in addition to being the predominant bacterial species reported in various international cohort studies of the human gut microbiome,[28–30] *F. prausnitzii* has also been reported to decrease in individuals

with various diseases, such as inflammatory bowel disease, diabetes, and chronic kidney disease;[18,31] thus, *F. prausnitzii* has recently attracted considerable attention as a next-generation health-promoting bacterium.[17,18] This accumulated evidence and our findings in the cross-sectional study brought us to selection and isolation of *F. prausnitzii* and our animal experiments, which revealed part of the previously unrecognized causal relationships between specific strains of *F. prausnitzii,* an MCI-related microbe, and cognitive function.

Pasteurized Fp14, to our surprise, reproducibly and significantly improved cognitive impairment in the PA test, which was not observed with live Fp14 (Figures 4 and S4). A similar phenomenon of the effect of pasteurization was observed in another next-generation, health-promoting, Gram-negative bacterium, *Akkermansia muciniphila*.[32] We speculate that pasteurization of Gram-negative bacteria enhances bioactivity by solubilizing extracellular components or by increasing the accessibility of membrane proteins. A limitation of using *F. prausnitzii* in humans is its high sensitivity to oxygen; thus, the efficacy gained by pasteurization would be a solution for use of strictly anaerobic health-promoting bacteria and provides insight into the potential properties of *F. prausnitzii* as a preventive intervention material.

The effect of pasteurized Fp14 on cognitive improvement was observed only in the PA test, not in the Y-maze test (Figure 4); the former evaluates long-term memory and the latter working memory. In terms of memory consolidation, the improvement of long-term memory includes improvement of working memory,[33] so the effect of pasteurized Fp14 on memory function seems to be obvious. Thus, no significant differences in the Y-maze test could be explained by the possibility that the effect of improvement of working memory by Fp14 was masked by other effects on brain functions, such as curiosity. Further research regarding other effects on brain functions is needed to reveal the overall mechanism of action of pasteurized Fp14. On the other hand, the effect of live Fp360 on cognitive improvement was observed in the PA test and the Y-maze test (Figure 4). The total number of specific orthologs that were found only in Fp360 was larger than that of Fp14 (Table S5), so we could speculate that the combination of specific orthologs found solely in Fp360 could potentially be related to its stronger efficacy compared with Fp14, but the specific mechanism of action of live Fp360 needs to be elucidated in further studies.

Whole-genome comparison of *F. prausnitzii* isolates and verification by metagenomics data revealed that some of the specific orthologs found in the effective strains were significantly higher in the healthy group than in the MCI group (Figures 5 and 6). One specific ortholog, OG0003536, which was shared only by Fp14 and Fp360, was significantly higher in the healthy group and predicted to be a transmembrane protein (Figures 5A, 6A, and 6B). It is well known that transmembrane proteins mediate multiple interactions between a bacterium and its environment,[34] so it is implied that this shared protein would be related to the effect of cognitive improvement with Fp14 and Fp360. However, given the differences in the effective form between Fp14 (pasteurized) and Fp360 (live), we speculate that combination of OG0003536 with specific orthologs that were identified exclusively in each strain would more deeply explain the effect of cognitive improvement.

Four specific orthologs that were found solely in Fp14 were significantly higher in the healthy group and surprisingly adjacent to each other (Figures 5B, 6A, and 6B). Two implications could be derived from these results. First, they were all predicted to be an extracellular protein or a transmembrane protein (Figure 5B), so one speculation is that pasteurization increases their accessibility to the host, solubilizes these proteins, or changes their conformation, which would lead to efficacy of Fp14 as a pasteurized form only. Second, 3 of the 4 specific orthologs were related to extracellular trehalose intake and trehalose metabolism (Figure 5B). Thus, another speculation is that the intracellular trehalose, trehalose 6-phosphate, or some sort of substances related to the trehalose metabolism, were excreted because of pasteurization and worked as the effective compound when administering pasteurized Fp14. This speculation is supported by a report showing that administration of trehalose to a Tg2576 mouse model of AD improved cognitive impairment.[35] Further research is needed to elucidate whether these extracellular and transmembrane proteins themselves or a combination of these proteins with trehalose-related metabolites or with OG0003536 would explain the efficacy of pasteurized Fp14.

Three specific orthologs that were found solely in Fp360 were significantly higher in the healthy group, and 2 of these 3 specific orthologs were related to cell division: K13052 (DivIC) and K09888 (ZapA) (Figures 6A and 6B; Table S5). The DivIC complex has been reported recently to have a large periplasmic domain and to direct peptidoglycan (PG) synthesis specific to the septum during cell division and start massive PG synthesis at the midcell for synthesis of the septum.[36] These PG structures and DivIC complex at the midcell appear only during cell division; therefore, we could speculate that the transient structures of PG and the DivIC complex are somehow related to the result that requires Fp360 to be in the live form to be efficacious. Further research is needed to reveal whether these cell division proteins themselves or combination of these proteins with OG0003536 would explain the efficacy of live Fp360.

We also analyzed whether known strains of *F. prausnitzii* have the specific orthologs that were shared by the effective strains Fp14 and Fp360. By comparison of the complete genomes of our isolated *F. prausnitzii* strains and three known strains (A2-165, S3L/3, and L2-6; Figure S3), we found 6 orthologs other than OG0000713 and OG0000772 among 8 orthologs (Figure 6) that were not shared by the aforementioned three strains. However, OG0000713 and OG0000772 (found solely in Fp360) were shared by the L2-6 strain. The detailed characteristics and the effect of the L2-6 strain on cognitive status is currently unknown, so further studies are needed to clarify whether L2-6 and Fp360 have the same efficacy profile and whether these bacteria are potent because of these shared specific orthologs.

The efficacy gained by pasteurization in Fp14 would be a feasible solution for use of strictly anaerobic health-promoting bacteria as a preventive intervention material. Thus, we focused on pasteurized Fp14 in metabolome and RNA-seq analyses of the hippocampus to explore the potential mechanism of efficacy. Regarding metabolome analysis, Fp14 significantly reduced thymine and 6mA and had a tendency to reduce suberic acid (Figure 7). These metabolites have been reported to be related to oxidative stress and mitochondrial function.[37–41] For example,

thymine glycol, an oxidized form of thymine, has been reported to accumulate in the brain of individuals with AD,[41], but thymine glycol was not measured in this metabolome analysis. Second, 6mA has been shown to increase during chronic restraint stress in mouse brains[39] and was related to the increase of reactive oxygen species (ROS) production in mitochondria.[37] Third, dicarboxylic acids (DCAs), such as suberic acid and azelaic acid, are formed by oxidative breakdown of unsaturated fatty acids and are known to modify mitochondrial function.[38] This evidence guided us to speculate that our results (Figure 7) exhibited a relationship between the efficacy of pasteurized Fp14 with oxidative stress and mitochondrial function. Regarding RNA-seq analysis, pasteurized Fp14 significantly reduced the transcripts of PACS-2 (Table S8). PACS-2 has been reported to play critical roles in mitochondrial dynamics and to be related to many diseases, including AD.[42] This evidence and our results (Table S8) also supported involvement of pasteurized Fp14 with mitochondrial function in the brain. Moreover, this potential involvement is also consistent with evidence showing that certain compounds restore cognitive function through reversion of the Aβ-induced reduction in mitochondrial mass and hippocampal neurogenesis in APP/PS1 mice.[43] Furthermore, evidence showing that trehalose ameliorates oxidative stress-mediated mitochondrial dysfunction[44] also supports the insights into the relationship between oxidative stress, mitochondrial function, and the efficacy of pasteurized Fp14, whose specific orthologs are related to trehalose metabolism (Figure 5B). Further studies are needed to elucidate the detailed involvement of pasteurized Fp14 in oxidative stress and mitochondrial function.

Comparison of gut microbial composition of healthy, MCI, and AD groups led us to selection and isolation of *F. prausnitzii* as a potential MCI-preventive microbe for gut microbiome-based intervention in MCI. We next demonstrated part of the causal relationship between the strains of *F. prausnitzii*, an MCI-related microbe, and cognitive function by administration of isolated *F. prausnitzii* strains to an AD mouse model. The efficacy gained by pasteurization highlighted the potential for resolving the burden of using health-promoting bacteria, *F. prausnitzii*, as a preventive intervention material in humans despite its high sensitivity to oxygen. Moreover, whole-genome comparison and metagenome shotgun sequencing revealed specific orthologs that were found only in the effective *F. prausnitzii* strains and were significantly abundant in the healthy group compared with the MCI group, which implied a relationship of these specific orthologs with MCI and part of the mechanism of efficacy in different bacterial forms (pasteurized or live). In addition, metabolome and RNA-seq analyses exhibited a relationship between the efficacy of pasteurized Fp14, oxidative stress, and mitochondrial function in the brain. Thus, our findings highlight the characteristics of *F. prausnitzii* strains with these specific orthologs as candidates for gut microbiome-based intervention in Alzheimer's-type dementia.

LIMITATIONS OF THE STUDY

The following limitations should be considered. First, although the relationship between specific microbes and cognitive function was found based on a cross-sectional study, age-matched validation cohorts with large sample sizes, longitudinal cohorts, and intervention trials at the human level are needed to confirm these results. Second, although the mouse model in this study has symptomatic and pathophysiological similarities to human AD and could be useful for evaluating candidates for intervention in AD through behavioral experiments (Methods details), the model does not reflect the long-term pathological progress of AD, such as Aβ deposition. Thus, investigating the efficacy of pasteurized Fp14 and live Fp360 on Aβ and tau deposition in other AD mouse models would be of interest in future studies. Third, although specific orthologs and potentially mechanistically related metabolites or transcripts were found, further investigations are required to elucidate whether these specific orthologs and potentially mechanistically related metabolites or transcripts are related to mediation of cognitive improvement of pasteurized Fp14 and live Fp360.

STAR★METHODS

Detailed methods are provided in the online version of this paper and include the following:

- KEY RESOURCES TABLE
- RESOURCE AVAILABILITY
  - Lead contact
  - Materials availability
  - Data and code availability
- EXPERIMENTAL MODEL AND SUBJECT DETAILS
  - Participants
  - Animals
- METHOD DETAILS
  - DNA extraction
  - 16S rRNA gene sequencing
  - Gut microbial composition analysis
  - Isolation of F. prausnitzii
  - Phylogenetic analysis of F. prausnitzii
  - Preparation of bacterial samples
  - Intracerebroventricular (i.c.v.) injection
  - Behavioral tests
  - Whole-genome comparison
  - Metagenome shotgun sequencing
  - Quality control of whole-genome shotgun reads
  - Calculation of ortholog abundance
  - Metabolome Analysis
  - RNA-seq Analysis
- QUANTIFICATION AND STATISTICAL ANALYSIS

SUPPLEMENTAL INFORMATION

Supplemental information can be found online at https://doi.org/10.1016/j.xcrm.2021.100398.

ACKNOWLEDGMENTS

We thank all study participants and their families; S. Seino, T. Ikeuchi, Y. Fujiwara, and other members of the Tokyo Metropolitan Institute of Gerontology (TMIG); H. Murasawa and other members of Nihon Bioresearch Inc.; Y. Kotani, M. Toba, S. Inoue, C.R. Quach, M. Kishimoto, K. Higuchi, H. Inose, and other members of Otsuka Pharmaceutical Co., Ltd.; and H. Mori, K. Kurokawa, H. Tsuchikura, and other members of the Tokyo Institute of Technology (Tokyo

Tech). This study was conducted under a contract between TMIG, Tokyo Tech, and Otsuka Pharmaceutical Co., Ltd. and supported by Grants-In-Aid for Scientific Research (B) (JP26310111) from the Ministry of Education, Culture, Sports, Science and Technology, Japan; Otsuka Pharmaceutical Co., Ltd., Japan; and the Tokyo Tech Fund, Japan.

AUTHOR CONTRIBUTIONS

Conceptualization, S.S., A.U., K.U., N.K., and T.Y.; investigation, A.U., H.S., S.S., Y.T., S.T., K.U., T. Kariya, T. Kawahara, and Y.K.; formal analysis, A.U., Y.T., H.S., T. Kariya, T. Kawahara, and Y.K.; resources, S.S., A.K., A.U., and T.Y.; data curation, A.U., Y.T., H.S., T. Kariya, and T.Y.; software, A.U., H.S., and T.Y.; writing – original draft, A.U.; writing – review & editing, A.U. and T.Y.; funding acquisition, S.S., A.K., N.K., and T.Y.

DECLARATION OF INTERESTS

The Tokyo Institute of Technology and Otsuka Pharmaceutical Co., Ltd. are currently applying for a patent dealing with the use of *F. prausnitzii* and its components for prevention, diagnosis, and treatment of dementia. The inventors are A.K., S.S., T.Y., S.T., K.U., A.U., T. Kariya, T. Kawahara, Y.K., and N.K. T.Y. is a founder of Metabologenomics and Metagen Therapeutics. The companies focus on design and control of the gut environment for human health but had no control over the experiments, interpretations, writing, or publication of this work. The terms of these arrangements are being managed by the Tokyo Metropolitan Institute of Gerontology; Otsuka Pharmaceutical Co., Ltd.; and the Tokyo Institute of Technology in accordance with their conflict of interest policies.

Received: February 17, 2021
Revised: May 29, 2021
Accepted: August 18, 2021
Published: September 14, 2021

REFERENCES

1. World Health Organization (2017). Global action plan on the public health response to dementia 2017–2025. https://www.who.int/mental_health/neurology/dementia/action_plan_2017_2025/en/.
2. Crous-Bou, M., Minguillón, C., Gramunt, N., and Molinuevo, J.L. (2017). Alzheimer's disease prevention: from risk factors to early intervention. Alzheimers Res. Ther. *9*, 71.
3. Jack, C.R., Jr., Knopman, D.S., Jagust, W.J., Petersen, R.C., Weiner, M.W., Aisen, P.S., Shaw, L.M., Vemuri, P., Wiste, H.J., Weigand, S.D., et al. (2013). Tracking pathophysiological processes in Alzheimer's disease: an updated hypothetical model of dynamic biomarkers. Lancet Neurol. *12*, 207–216.
4. Haeberlein, S.B., von Hehn, C., Tian, Y., Chalkias, S., Muralidharan, K.K., Chen, T., Wu, S., Li, J., Skordos, L., Nisenbaum, L., et al. (2020). EMERGE and ENGAGE Topline Results: Two Phase 3 Studies to Evaluate Aducanumab in Patients With Early Alzheimer's Disease. In: AAN Science Highlights. https://investors.biogen.com/static-files/ccb1c817-6d20-4987-b9dc-bcfa96829691.
5. Long, J.M., and Holtzman, D.M. (2019). Alzheimer Disease: An Update on Pathobiology and Treatment Strategies. Cell *179*, 312–339.
6. Cryan, J.F., O'Riordan, K.J., Cowan, C.S.M., Sandhu, K.V., Bastiaanssen, T.F.S., Boehme, M., Codagnone, M.G., Cussotto, S., Fulling, C., Golubeva, A.V., et al. (2019). The microbiota-gut-brain axis. Physiol. Rev. *99*, 1877–2013.
7. Fang, P., Kazmi, S.A., Jameson, K.G., and Hsiao, E.Y. (2020). The Microbiome as a Modifier of Neurodegenerative Disease Risk. Cell Host Microbe *28*, 201–222.
8. Saji, N., Murotani, K., Hisada, T., Tsuduki, T., Sugimoto, T., Kimura, A., Niida, S., Toba, K., and Sakurai, T. (2019). The relationship between the gut microbiome and mild cognitive impairment in patients without dementia: a cross-sectional study conducted in Japan. Sci. Rep. *9*, 19227.
9. Liu, P., Wu, L., Peng, G., Han, Y., Tang, R., Ge, J., Zhang, L., Jia, L., Yue, S., Zhou, K., et al. (2019). Altered microbiomes distinguish Alzheimer's disease from amnestic mild cognitive impairment and health in a Chinese cohort. Brain Behav. Immun. *80*, 633–643.
10. Takeda, S., Sato, N., Niisato, K., Takeuchi, D., Kurinami, H., Shinohara, M., Rakugi, H., Kano, M., and Morishita, R. (2009). Validation of Abeta1-40 administration into mouse cerebroventricles as an animal model for Alzheimer disease. Brain Res. *1280*, 137–147.
11. Hiramatsu, M., Takiguchi, O., Nishiyama, A., and Mori, H. (2010). Cilostazol prevents amyloid β peptide(25-35)-induced memory impairment and oxidative stress in mice. Br. J. Pharmacol. *161*, 1899–1912.
12. Meunier, J., Ieni, J., and Maurice, T. (2006). The anti-amnesic and neuroprotective effects of donepezil against amyloid beta25-35 peptide-induced toxicity in mice involve an interaction with the σ1 receptor. Br. J. Pharmacol. *149*, 998–1012.
13. Gavriel, Y., Rabinovich-Nikitin, I., Ezra, A., Barbiro, B., and Solomon, B. (2020). Subcutaneous Administration of AMD3100 into Mice Models of Alzheimer's Disease Ameliorated Cognitive Impairment, Reduced Neuroinflammation, and Improved Pathophysiological Markers. J. Alzheimers Dis. *78*, 653–671.
14. Yang, R., Chen, L., Wang, H., Xu, B., Tomimoto, H., and Chen, L. (2012). Anti-amnesic effect of neurosteroid PREGS in Aβ25-35-injected mice through σ1 receptor- and α7nAChR-mediated neuroprotection. Neuropharmacology *63*, 1042–1050.
15. Song, L., Pei, L., Hu, L., Pan, S., Xiong, W., Liu, M., Wu, Y., Shang, Y., and Yao, S. (2018). Death-associated protein kinase 1 mediates interleukin-1β production through regulating inlfammasome activation in Bv2 microglial cells and mice. Sci. Rep. *8*, 9930.
16. Villard, V., Espallergues, J., Keller, E., Alkam, T., Nitta, A., Yamada, K., Nabeshima, T., Vamvakides, A., and Maurice, T. (2009). Antiamnesic and neuroprotective effects of the aminotetrahydrofuran derivative ANAVEX1-41 against amyloid beta(25-35)-induced toxicity in mice. Neuropsychopharmacology *34*, 1552–1566.
17. Langella, P., Guarner, F., and Martín, R. (2019). Editorial: Next-generation probiotics: From commensal bacteria to novel drugs and food supplements. Front. Microbiol. *10*, 1973.
18. Martín, R., Bermúdez-Humarán, L.G., and Langella, P. (2018). Searching for the bacterial effector: The example of the multi-skilled commensal bacterium Faecalibacterium prausnitzii. Front. Microbiol. *9*, 346.
19. Xu, C., Zhu, H., and Qiu, P. (2019). Aging progression of human gut microbiota. BMC Microbiol. *19*, 236.
20. Zhang, X., Wang, Y., Liu, W., Wang, T., Wang, L., Hao, L., Ju, M., and Xiao, R. (2021). Diet quality, gut microbiota, and microRNAs associated with mild cognitive impairment in middle-aged and elderly Chinese population. Am. J. Clin. Nutr. *114*, 429–440.
21. Nagpal, R., Neth, B.J., Wang, S., Craft, S., and Yadav, H. (2019). Modified Mediterranean-ketogenic diet modulates gut microbiome and short-chain fatty acids in association with Alzheimer's disease markers in subjects with mild cognitive impairment. EBioMedicine *47*, 529–542.
22. Kasai, C., Sugimoto, K., Moritani, I., Tanaka, J., Oya, Y., Inoue, H., Tameda, M., Shiraki, K., Ito, M., Takei, Y., and Takase, K. (2016). Comparison of human gut microbiota in control subjects and patients with colorectal carcinoma in adenoma: Terminal restriction fragment length polymorphism and next-generation sequencing analyses. Oncol. Rep. *35*, 325–333.
23. Fujimoto, T., Imaeda, H., Takahashi, K., Kasumi, E., Bamba, S., Fujiyama, Y., and Andoh, A. (2013). Decreased abundance of Faecalibacterium prausnitzii in the gut microbiota of Crohn's disease. J. Gastroenterol. Hepatol. *28*, 613–619.

24. Gaulke, C.A., and Sharpton, T.J. (2018). The influence of ethnicity and geography on human gut microbiome composition. Nat. Med. *24*, 1495–1496.
25. Vogt, N.M., Kerby, R.L., Dill-McFarland, K.A., Harding, S.J., Merluzzi, A.P., Johnson, S.C., Carlsson, C.M., Asthana, S., Zetterberg, H., Blennow, K., et al. (2017). Gut microbiome alterations in Alzheimer's disease. Sci. Rep. *7*, 13537.
26. Haran, J.P., Bhattarai, S.K., Foley, S.E., Dutta, P., Ward, D.V., Bucci, V., and McCormick, B.A. (2019). Alzheimer's Disease Microbiome Is Associated with Dysregulation of the Anti-Inflammatory P-Glycoprotein Pathway. MBio *10*, 1–14.
27. Ling, Z., Zhu, M., Yan, X., Cheng, Y., Shao, L., Liu, X., Jiang, R., and Wu, S. (2021). Structural and Functional Dysbiosis of Fecal Microbiota in Chinese Patients With Alzheimer's Disease. Front. Cell Dev. Biol. *8*, 634069.
28. Liu, H., Pan, L.L., Lv, S., Yang, Q., Zhang, H., Chen, W., Lv, Z., and Sun, J. (2019). Alterations of gut microbiota and blood lipidome in gestational diabetes mellitus with hyperlipidemia. Front. Physiol. *10*, 1015.
29. Shikany, J.M., Demmer, R.T., Johnson, A.J., Fino, N.F., Meyer, K., Ensrud, K.E., Lane, N.E., Orwoll, E.S., Kado, D.M., Zmuda, J.M., and Langsetmo, L.; Osteoporotic Fractures in Men (MrOS) Research Group (2019). Association of dietary patterns with the gut microbiota in older, community-dwelling men. Am. J. Clin. Nutr. *110*, 1003–1014.
30. Mueller, S., Saunier, K., Hanisch, C., Norin, E., Alm, L., Midtvedt, T., Cresci, A., Silvi, S., Orpianesi, C., Verdenelli, M.C., et al. (2006). Differences in fecal microbiota in different European study populations in relation to age, gender, and country: a cross-sectional study. Appl. Environ. Microbiol. *72*, 1027–1033.
31. Leylabadlo, H.E., Ghotaslou, R., Feizabadi, M.M., Farajnia, S., Moaddab, S.Y., Ganbarov, K., Khodadadi, E., Tanomand, A., Sheykhsaran, E., Yousefi, B., and Kafil, H.S. (2020). The critical role of Faecalibacterium prausnitzii in human health: An overview. Microb. Pathog. *149*, 104344.
32. Plovier, H., Everard, A., Druart, C., Depommier, C., Van Hul, M., Geurts, L., Chilloux, J., Ottman, N., Duparc, T., Lichtenstein, L., et al. (2017). A purified membrane protein from Akkermansia muciniphila or the pasteurized bacterium improves metabolism in obese and diabetic mice. Nat. Med. *23*, 107–113.
33. Meeter, M., and Murre, J.M.J. (2004). Consolidation of long-term memory: evidence and alternatives. Psychol. Bull. *130*, 843–857.
34. Sberro, H., Fremin, B.J., Zlitni, S., Edfors, F., Greenfield, N., Snyder, M.P., Pavlopoulos, G.A., Kyrpides, N.C., and Bhatt, A.S. (2019). Large-Scale Analyses of Human Microbiomes Reveal Thousands of Small, Novel Genes. Cell *178*, 1245–1259.e14.
35. Portbury, S.D., Hare, D.J., Sgambelloni, C., Perronnes, K., Portbury, A.J., Finkelstein, D.I., and Adlard, P.A. (2017). Trehalose Improves Cognition in the Transgenic Tg2576 Mouse Model of Alzheimer's Disease. J. Alzheimers Dis. *60*, 549–560.
36. Monteiro, J.M., Pereira, A.R., Reichmann, N.T., Saraiva, B.M., Fernandes, P.B., Veiga, H., Tavares, A.C., Santos, M., Ferreira, M.T., Macário, V., et al. (2018). Peptidoglycan synthesis drives an FtsZ-treadmilling-independent step of cytokinesis. Nature *554*, 528–532.
37. Hao, Z., Wu, T., Cui, X., Zhu, P., Tan, C., Dou, X., Hsu, K.W., Lin, Y.T., Peng, P.H., Zhang, L.S., et al. (2020). N^6-Deoxyadenosine Methylation in Mammalian Mitochondrial DNA. Mol. Cell *78*, 382–395.e8.
38. Castor, K.J., Shenoi, S., Edminster, S.P., Tran, T., King, K.S., Chui, H., Pogoda, J.M., Fonteh, A.N., and Harrington, M.G. (2020). Urine dicarboxylic acids change in pre-symptomatic Alzheimer's disease and reflect loss of energy capacity and hippocampal volume. PLoS ONE *15*, e0231765.
39. Yao, B., Cheng, Y., Wang, Z., Li, Y., Chen, L., Huang, L., Zhang, W., Chen, D., Wu, H., Tang, B., and Jin, P. (2017). DNA N6-methyladenine is dynamically regulated in the mouse brain following environmental stress. Nat. Commun. *8*, 1122.
40. Zhao, L., Liu, Z., Jia, H., Feng, Z., Liu, J., and Li, X. (2015). Lipoamide acts as an indirect antioxidant by simultaneously stimulating mitochondrial biogenesis and phase II antioxidant enzyme systems in ARPE-19 cells. PLoS ONE *10*, e0128502.
41. Lovell, M.A., and Markesbery, W.R. (2007). Oxidative DNA damage in mild cognitive impairment and late-stage Alzheimer's disease. Nucleic Acids Res. *35*, 7497–7504.
42. Li, C., Li, L., Yang, M., Zeng, L., and Sun, L. (2020). PACS-2: A key regulator of mitochondria-associated membranes (MAMs). Pharmacol. Res. *160*, 105080.
43. Bartolome, F., de la Cueva, M., Pascual, C., Antequera, D., Fernandez, T., Gil, C., Martinez, A., and Carro, E. (2018). Amyloid β-induced impairments on mitochondrial dynamics, hippocampal neurogenesis, and memory are restored by phosphodiesterase 7 inhibition. Alzheimers Res. Ther. *10*, 24.
44. Tang, Q., Zheng, G., Feng, Z., Chen, Y., Lou, Y., Wang, C., Zhang, X., Zhang, Y., Xu, H., Shang, P., and Liu, H. (2017). Trehalose ameliorates oxidative stress-mediated mitochondrial dysfunction and ER stress via selective autophagy stimulation and autophagic flux restoration in osteoarthritis development. Cell Death Dis. *8*, e3081.
45. Bolyen, E., Rideout, J.R., Dillon, M.R., Bokulich, N.A., Abnet, C.C., Al-Ghalith, G.A., Alexander, H., Alm, E.J., Arumugam, M., Asnicar, F., et al. (2019). Reproducible, interactive, scalable and extensible microbiome data science using QIIME 2. Nat. Biotechnol. *37*, 852–857.
46. Kumar, S., Stecher, G., Li, M., Knyaz, C., and Tamura, K. (2018). MEGA X: Molecular evolutionary genetics analysis across computing platforms. Mol. Biol. Evol. *35*, 1547–1549.
47. Hunt, M., Silva, N.D., Otto, T.D., Parkhill, J., Keane, J.A., and Harris, S.R. (2015). Circlator: automated circularization of genome assemblies using long sequencing reads. Genome Biol. *16*, 294.
48. Parks, D.H., Imelfort, M., Skennerton, C.T., Hugenholtz, P., and Tyson, G.W. (2015). CheckM: assessing the quality of microbial genomes recovered from isolates, single cells, and metagenomes. Genome Res. *25*, 1043–1055.
49. Seemann, T. (2014). Prokka: rapid prokaryotic genome annotation. Bioinformatics *30*, 2068–2069.
50. Jones, P., Binns, D., Chang, H.Y., Fraser, M., Li, W., McAnulla, C., McWilliam, H., Maslen, J., Mitchell, A., Nuka, G., et al. (2014). InterProScan 5: genome-scale protein function classification. Bioinformatics *30*, 1236–1240.
51. Krogh, A., Larsson, B., von Heijne, G., and Sonnhammer, E.L.L. (2001). Predicting transmembrane protein topology with a hidden Markov model: application to complete genomes. J. Mol. Biol. *305*, 567–580.
52. Page, A.J., Cummins, C.A., Hunt, M., Wong, V.K., Reuter, S., Holden, M.T.G., Fookes, M., Falush, D., Keane, J.A., and Parkhill, J. (2015). Roary: rapid large-scale prokaryote pan genome analysis. Bioinformatics *31*, 3691–3693.
53. Emms, D.M., and Kelly, S. (2019). OrthoFinder: phylogenetic orthology inference for comparative genomics. Genome Biol. *20*, 238.
54. Buchfink, B., Xie, C., and Huson, D.H. (2015). Fast and sensitive protein alignment using DIAMOND. Nat. Methods *12*, 59–60.
55. Martin, M. (2011). Cutadapt removes adapter sequences from high-throughput sequencing reads. EMBnet. J. *17*, 10–12.
56. Langmead, B., and Salzberg, S.L. (2012). Fast gapped-read alignment with Bowtie 2. Nat. Methods *9*, 357–359.
57. Bray, N.L., Pimentel, H., Melsted, P., and Pachter, L. (2016). Near-optimal probabilistic RNA-seq quantification. Nat. Biotechnol. *34*, 525–527.
58. Kim, D., Paggi, J.M., Park, C., Bennett, C., and Salzberg, S.L. (2019). Graph-based genome alignment and genotyping with HISAT2 and HISAT-genotype. Nat. Biotechnol. *37*, 907–915.
59. Pertea, M., Pertea, G.M., Antonescu, C.M., Chang, T.C., Mendell, J.T., and Salzberg, S.L. (2015). StringTie enables improved reconstruction of a transcriptome from RNA-seq reads. Nat. Biotechnol. *33*, 290–295.
60. Li, H., Handsaker, B., Wysoker, A., Fennell, T., Ruan, J., Homer, N., Marth, G., Abecasis, G., and Durbin, R.; 1000 Genome Project Data Processing

Subgroup (2009). The Sequence Alignment/Map format and SAMtools. Bioinformatics *25*, 2078–2079.

61. Taniguchi, Y., Yoshida, H., Fujiwara, Y., Motohashi, Y., and Shinkai, S. (2012). A prospective study of gait performance and subsequent cognitive decline in a general population of older Japanese. J. Gerontol. A Biol. Sci. Med. Sci. *67*, 796–803.
62. Fujiwara, Y., Suzuki, H., Yasunaga, M., Sugiyama, M., Ijuin, M., Sakuma, N., Inagaki, H., Iwasa, H., Ura, C., Yatomi, N., et al. (2010). Brief screening tool for mild cognitive impairment in older Japanese: validation of the Japanese version of the Montreal Cognitive Assessment. Geriatr. Gerontol. Int. *10*, 225–232.
63. Berg, L. (1988). Clinical Dementia Rating (CDR). Psychopharmacol. Bull. *24*, 637–639.
64. Koyano, W., Shibata, H., Nakazato, K., Haga, H., and Suyama, Y. (1991). Measurement of competence: reliability and validity of the TMIG Index of Competence. Arch. Gerontol. Geriatr. *13*, 103–116.
65. Kitamura, A., Taniguchi, Y., Seino, S., Yokoyama, Y., Amano, H., Fujiwara, Y., and Shinkai, S. (2019). Combined effect of diabetes and frailty on mortality and incident disability in older Japanese adults. Geriatr. Gerontol. Int. *19*, 423–428.
66. Petersen, R.C. (2004). Mild cognitive impairment as a diagnostic entity. J. Intern. Med. *256*, 183–194.
67. American Psychiatric Association (1994). Diagnostic and statistical manual of mental disorders: DSM-IV (American Psychiatric Association).
68. Ichinose, T., Murasawa, H., Ishijima, T., Okada, S., Abe, K., Matsumoto, S., Matsui, T., and Furuya, S. (2020). Tyr-Trp administration facilitates brain norepinephrine metabolism and ameliorates a short-term memory deficit in a mouse model of Alzheimer's disease. PLoS ONE *15*, e0232233.
69. Yachida, S., Mizutani, S., Shiroma, H., Shiba, S., Nakajima, T., Sakamoto, T., Watanabe, H., Masuda, K., Nishimoto, Y., Kubo, M., et al. (2019). Metagenomic and metabolomic analyses reveal distinct stage-specific phenotypes of the gut microbiota in colorectal cancer. Nat. Med. *25*, 968–976.
70. Mori, H., Maruyama, F., Kato, H., Toyoda, A., Dozono, A., Ohtsubo, Y., Nagata, Y., Fujiyama, A., Tsuda, M., and Kurokawa, K. (2014). Design and experimental application of a novel non-degenerate universal primer set that amplifies prokaryotic 16S rRNA genes with a low possibility to amplify eukaryotic rRNA genes. DNA Res. *21*, 217–227.
71. Tsuchida, S., Kitahara, M., Nguema, P.P.M., Norimitsu, S., Fujita, S., Yamagiwa, J., Ngomanda, A., Ohkuma, M., and Ushida, K. (2014). Lactobacillus gorillae sp. nov., isolated from the faeces of captive and wild western lowland gorillas (Gorilla gorilla gorilla). Int. J. Syst. Evol. Microbiol. *64*, 4001–4006.
72. Turner, S., Pryer, K.M., Miao, V.P.W., and Palmer, J.D. (1999). Investigating deep phylogenetic relationships among cyanobacteria and plastids by small subunit rRNA sequence analysis. J. Eukaryot. Microbiol. *46*, 327–338.
73. Miyake, T., Watanabe, K., Watanabe, T., and Oyaizu, H. (1998). Phylogenetic analysis of the genus Bifidobacterium and related genera based on 16S rDNA sequences. Microbiol. Immunol. *42*, 661–667. https://www.ncbi.nlm.nih.gov/pubmed/9858460.
74. Lopez-Siles, M., Khan, T.M., Duncan, S.H., Harmsen, H.J.M., Garcia-Gil, L.J., and Flint, H.J. (2012). Cultured representatives of two major phylogroups of human colonic Faecalibacterium prausnitzii can utilize pectin, uronic acids, and host-derived substrates for growth. Appl. Environ. Microbiol. *78*, 420–428.
75. Frozza, R.L., Horn, A.P., Hoppe, J.B., Simão, F., Gerhardt, D., Comiran, R.A., and Salbego, C.G. (2009). A comparative study of β-amyloid peptides Abeta1-42 and Abeta25-35 toxicity in organotypic hippocampal slice cultures. Neurochem. Res. *34*, 295–303.
76. Zhang, Z., Guan, J., Jiang, Z., Yang, Y., Liu, J., Hua, W., Mao, Y., Li, C., Lu, W., Qian, J., and Zhan, C. (2019). Brain-targeted drug delivery by manipulating protein corona functions. Nat. Commun. *10*, 3561.
77. Smith, A.K., and Klimov, D.K. (2019). De novo aggregation of Alzheimer's Aβ25-35 peptides in a lipid bilayer. Sci. Rep. *9*, 7161.
78. Makino, M., Takahashi-Ito, K., Murasawa, H., Pawlak, A., Kashimoto, Y., and Kitano, Y. (2019). Memantine ameliorates learning and memory disturbance and the behavioral and psychological symptoms of dementia in thiamine-deficient mice. Pharmacol. Biochem. Behav. *183*, 6–13.
79. Sugimoto, M., Wong, D.T., Hirayama, A., Soga, T., and Tomita, M. (2010). Capillary electrophoresis mass spectrometry-based saliva metabolomics identified oral, breast and pancreatic cancer-specific profiles. Metabolomics *6*, 78–95.
80. Pertea, M., Kim, D., Pertea, G.M., Leek, J.T., and Salzberg, S.L. (2016). Transcript-level expression analysis of RNA-seq experiments with HISAT, StringTie and Ballgown. Nat. Protoc. *11*, 1650–1667.

STAR★METHODS

KEY RESOURCES TABLE

REAGENT or RESOURCE	SOURCE	IDENTIFIER
Bacterial and virus strains		
F. prausnitzii Fp1	This paper	N/A
F. prausnitzii Fp4	This paper	N/A
F. prausnitzii Fp14	This paper	N/A
F. prausnitzii Fp28	This paper	N/A
F. prausnitzii Fp40	This paper	N/A
F. prausnitzii Fp45	This paper	N/A
F. prausnitzii Fp77	This paper	N/A
F. prausnitzii Fp137	This paper	N/A
F. prausnitzii Fp360	This paper	N/A
F. prausnitzii Fp944	This paper	N/A
F. prausnitzii Fp1043	This paper	N/A
F. prausnitzii Fp1160	This paper	N/A
Biological samples		
Healthy, MCI, and AD fecal samples	This paper	N/A
Chemicals, peptides, and recombinant proteins		
Nextera Index Kit	Ilumina	FC-131-1002
TruSeq Nano DNA Library Prep Kit	Ilumina	FC-121-4003
Deposited data		
16S rRNA sequencing (Japanese, n = 43)	This paper	NCBI Sequence Read Archive: PRJNA679347
Whole-genome sequencing (12 isolates of *F. prausnitzii*)	This paper	NCBI Genome: PRJNA679348
Whole-metagenome shotgun sequencing (Japanese, n = 43)	This paper	NCBI Sequence Read Archive: PRJNA679346
RNA sequencing of mouse hippocampus (n = 51)	This paper	NCBI Sequence Read Archive: PRJNA735905
Genome of *F.prausnitzii* A2-165	GenBank	GCA_010509575.1
Genome of *F.prausnitzii* S3L/3	GenBank	GCA_000209855.1
Genome of *F.prausnitzii* L2-6	GenBank	GCA_000210735.1
Experimental models: Organisms/strains		
ddY mice	Japan SLC, Inc.	RRID: MGI:5558113
Software and Algorithms		
QIIME2	Bolyen et al.[45]	https://qiime2.org/
MEGA	Kumar et al., 2018[46]	https://www.megasoftware.net/
Circlator	Hunt et al.[47]	http://sanger-pathogens.github.io/circlator/
CheckM	Parks et al.[48]	http://ecogenomics.github.io/CheckM/
Prokka	Seemann[49]	https://vicbioinformatics.com/software.prokka.shtml
InterProScan	Jones et al.[50]	http://www.ebi.ac.uk/interpro/search/sequence/
TMHMM	Krogh et al.[51]	http://www.cbs.dtu.dk/services/TMHMM/
Roary	Page et al.[52]	https://sanger-pathogens.github.io/Roary/

(*Continued on next page*)

CellPress
OPEN ACCESS

Continued

REAGENT or RESOURCE	SOURCE	IDENTIFIER
Orthofinder	Emms and Kelly[53]	https://github.com/davidemms/OrthoFinder
DIAMOND	Buchfink et al.[54]	https://github.com/bbuchfink/diamond
Cutadapt	Martin[55]	https://cutadapt.readthedocs.io/en/stable/
Bowtie2	Langmead and Salzberg[56]	http://bowtie-bio.sourceforge.net/bowtie2/index.shtml
Kallisto	Bray et al.[57]	https://pachterlab.github.io/kallisto/about
Hisat2	Kim et al.[58]	https://daehwankimlab.github.io/hisat2/
StringTie	Pertea et al.[59]	http://ccb.jhu.edu/software/stringtie/
SAMtools	Li et al.[60]	http://samtools.sourceforge.net/

RESOURCE AVAILABILITY

Lead contact

Further information and requests for resources and reagents should be directed to and will be fulfilled by the Lead Contact, Takuji Yamada (takuji@bio.titech.ac.jp).

Materials availability

There are restrictions to the availability of the *F. prausnitzii* strains because of a patent filed on the invention and materials related to this paper. *F. prausnitzii* strains in this paper will be made available upon request, but we may require a payment and/or a completed Materials Transfer Agreement if there is a potential for commercial application.

Data and code availability

The sequencing data reported in this paper are available from NCBI Sequence Read Archive (SRA) or NCBI Genome BioProject PRJNA679346, PRJNA679347 PRJNA679348, PRJNA735905. Any additional information required to reanalyze the data reported in this work paper is available from the Lead Contact upon request.

EXPERIMENTAL MODEL AND SUBJECT DETAILS

Participants

The flow diagram of the selection of the subjects and the characteristics of the selected subjects are shown in Figure S1, Table S1 and Table S3. Subjects of the healthy group (n = 21, % female = 61.9) and the MCI group (n = 15, % female = 60.0) aged 65 years or older were recruited from the participants in longitudinal studies of aging and health at the town of Kusatsu, Gunma Prefecture, Japan, in 2015.[61] Subjects in the AD group (n = 7, % female = 71.4) aged 65 years or older were recruited at Kusatsu from those (n = 5) who lived in a local nursing home in Kusatsu, from those (n = 26) who used public nursing services in Kusatsu, and from those (n = 12) who participated in longitudinal studies of aging and health at the town of Kusatsu, Gunma Prefecture. Participants in the present study were excluded if they (1) used antibiotics in the previous two weeks prior to fecal sampling, (2) underwent GI tract surgery in the past half year, (3) had a history of GI disorders such as inflammatory bowel disease, inflammatory bowel syndrome, GI cancer or gastrostomy, (4) had a history of diabetes, or (5) had a history of any significant neurologic or psychiatric diseases other than AD. This study was conducted in accordance with the declaration of Helsinki. Written informed consent was obtained from all participants after they were provided with a detailed explanation of the study protocol, which was approved (Approval No. 1548 in 2015) by the ethics committee of the Tokyo Metropolitan Institute of Gerontology (TMIG). Sample size was not estimated as we regarded this cross-sectional study as an observational study, not an interventional study. We aimed to find candidates for gut microbiome-based intervention and opted to confirm the result by the examination using animal model.

Participants underwent the following geriatric assessments: (1) Mini-Mental State Examination (MMSE), Montreal Cognitive Assessment Japanese version (MoCA-J),[62] and Clinical Dementia Rating (CDR)[63] to evaluate comprehensive cognitive function, (2) TMIG-Index of Competence to evaluate basic Activities of Daily Living (ADL),[64] (3) a medical interview to confirm medical history and current medication use, and (4) health checkup data, i.e., the results of blood tests and blood pressure tests as previously described.[65]

Participants were divided into three groups: healthy, MCI and AD. The healthy group and the MCI group were diagnosed based on Petersen's criteria.[66] Participants were diagnosed with healthy if they had (1) no memory complaints by the participants, (2) a MoCA-J score of 26 or over, (3) an MMSE score of 24 or over, (4) a CDR score of 0, (5) no impairment in five basic ADLs (ADL = 5), and (6) no neurologic or psychiatric disease.

Participants were diagnosed with MCI if they had (1) memory complaints by the participants, family members or physicians in the previous 6 months, (2) a MoCA-J score of 25 or under, (3) an MMSE score of 24 or over, (4) a CDR score of 0.5, (5) no impairment in five basic ADLs (ADL = 5), and (6) no neurologic or psychiatric disease.

Participants were diagnosed with AD by doctors based on the cognitive assessment result (MMSE), the blood test result, and the Diagnostic and Statistical Manual of Mental Disorders (DSM)-IV criteria.[67] Briefly, the following six criteria of DSM-IV were assessed based on the medical interviews and questionnaires on the participants and the nurses, and the review of the medical records: (I) the development of multiple cognitive deficits was manifested by both memory impairment and one (or more) of the following cognitive disturbances: aphasia, apraxia, agnosia, or disturbances in executive functioning; (II) the cognitive deficits in criteria (I) caused significant impairment in social or occupational functioning and represented a significant decline from a previous level of functioning; (III) the course was characterized by gradual onset and continuing cognitive decline; (IV) the cognitive deficits in criteria (I) were not due to any of the following: other central nervous system conditions that cause progressive deficits in memory and cognition, systemic conditions that are known to cause dementia, or substance-induced conditions; (V) the deficits did not occur exclusively during the course of a delirium; and (VI) the disturbance was not better accounted for by another Axis I disorder. Brain imaging was not performed when we used the DSM-IV criteria in this study. If we used other criteria, such as the National Institute of Neurological and Communicative Disorders and Stroke & the Alzheimer's Disease and Related Disorders Association (NINCDS-ADRDA), the AD participants in this study were categorized into possible AD or probable AD.

We isolated *F. prausnitzii* strains from the feces of healthy (Fp1, Fp4, Fp14, Fp28, Fp40, Fp45, Fp77, Fp137 and Fp360) and MCI (Fp944) volunteers who participated in our cross-sectional study under completely anaerobic conditions. Written informed consent was obtained from all participants after they were provided with a detailed explanation of the study protocol, which was approved (Approval No. 1042 in 2016) by the ethics committee of TMIG. We also isolated two *F. prausnitzii* strains (Fp1043 and Fp1160) from the feces of a healthy volunteer (n = 1, 30 years old). Written informed consent was obtained after the detailed explanation of the study protocol, which was approved (Approval No. ORQS-007 in 2016) by the ethics committee of Otsuka Pharmaceutical Co., Ltd.

Animals

Male 7-week-old ddY mice (Japan SLC, Inc., Japan) were given MF diet (Oriental Yeast Co., Ltd., Japan) and water *ad libitum*. Mice were housed in a specific pathogen-free controlled environment with a 12 h light/12 h dark cycle and constant temperature (25 °C) with under 4 mice per cage as previously described with some modifications.[68] All animal experiments were approved by the Animal Care and Use Committee of Nihon Bioresearch Inc., accredited by the Health Science Center for Accreditation of Laboratory Animal Care and Use of the Japan Health Sciences Foundation. All animal experimental procedures were performed in accordance with the recommendations in the Guide for the Care and Use of Laboratory Animals of the National Institutes of Health. Mice were daily administered 0.3 mL of live *F. prausnitzii,* pasteurized bacterial solution (the McFarland score of 20-fold dilution samples was 0.6, the corresponding bacterial concentration was 1.1×10^9 bacteria / animal, which was calculated based on the manual of our density measurement equipment (DENSIMAT, BioMérieux, France)) or culture supernatant by oral gavage for 11 days, starting 2 days before the Aβ injection (we confirmed that 0.3 mL gavage was below 10 mL/kg/day, the recommended max volume determined by mouse weight). Regarding the positive control group, mice were orally administered donepezil hydrochloride (0.5 mg/kg/day; Wako Chemicals, Japan). Regarding the sham-operation group and the vehicle group (Aβ-injected mice), mice were orally administered 0.3 mL of saline. Regarding the medium-administered group mice, mice were orally administered with 0.3 mL of LYBHI. No adverse effects were observed during the animal experiments. The number of biological replicates was 9 in Figure 3, 12 in Figure 4 and 12 in Figure S4

METHOD DETAILS

DNA extraction

Stool samples were collected immediately after defecation. Stool samples were mixed with 100 mM Tris-HCl (pH 9), 40 mM EDTA, 4 M guanidine thiocyanate, and 0.001% bromothymol, and frozen at −80°C until further analysis. DNA was extracted from the frozen fecal samples with the bead-beating method as previously described[69] using a GNOME DNA Isolation Kit (MP Biomedicals, USA). DNA quality was assessed with an Agilent 4200 Tape Station (Agilent Technologies, USA). After final precipitation, the DNA samples were resuspended in TE buffer and stored at −80°C before further analysis.

16S rRNA gene sequencing

Barcoded amplicon libraries targeting an ~300 bp fragment of the V3-V4 region of the bacterial 16S rRNA gene were prepared using the universal primer pair 342F-806R[70] and Nextera XT Index Kit (Illumina, CA, USA). The PCR products were individually concentrated and purified using a 2% E-Gel SizeSelect agarose gel (Thermo Fisher Scientific) and AMPure XP beads (Beckmann Coulter, Brea, CA, USA). Final libraries were quantified using the Quant-iT dsDNA HS Assay kit (Thermo Fisher Scientific, USA) and the High Sensitivity DNA kit (Agilent Technologies). Sequencing was carried out with a MiSeq sequencer (Illumina), according to the manufacturer's instructions.

Gut microbial composition analysis

The sequenced reads were then processed using the QIIME 2™ pipeline (v 2019.7.0).[45] In brief, reads were denoised by the dada2 plugin, and taxonomic classification was performed using the GTDB (Genome Taxonomy Database, date: July 2019;

https://gtdb.ecogenomic.org/#) and the Naive-Bayes classifier. One sample with less than 10,000 sequencing reads was removed (Table S3). Genera, which were detected in a sole sample or only in two samples, and genera with a mean abundance below 0.01% were filtered. Differential abundance analysis was performed using ALDEx2 library in R (v3.6.3) (Figure 2). Principal coordinates analysis (PCoA) with Bray-Curtis distance and the calculation of Shannon-Wiener alpha-diversity index were performed by QIIME 2^{TM} and R (v3.6.3) to examine the difference of overall microbiome characteristics between groups (Figures 2 and S2).

Isolation of F. prausnitzii

Freshly emitted fecal samples were serially diluted 10^{-5} to 10^{-7} with dilution medium (per 1 L; K_2HPO_4 0.45 g, NaCl 0.9 g, $(NH_4)_2SO_4$ 0.9 g, KH_2PO_4 0.45 g, $MgSO_4 \cdot 7H_2O$ 0.19 g, $CaCl_2 \cdot 2H_2O$ 0.12 g, Na_2CO_3 80 g, agar 0.5 g, resazurin 1 mg, L-cysteine·HCl·H_2O 0.5 g) in anaerobic chambers (N_2 = 90%, CO_2 = 5%, H_2 = 5%). The diluents were then plated on either YCFA agar medium (per 1 L; casitone 10 g, yeast extract 2.5 g, $NaHCO_3$ 4 g, glucose 4.5 g, hemin 10 mg, Tween 80 1 mL, agar 15 g, K_2HPO_4 0.45 g, KH_2PO_4 0.45 g, $(NH_4)_2SO_4$ 0.9 g, NaCl 0.9 g, $MgSO_4 \cdot 7H_2O$ 0.09 g, $CaCl_2 \cdot 2H_2O$ 0.12 g, L-cysteine·HCl·H_2O 1.4 g, acetic acid 53 μL, propionic acid 19 μL, n-valeric acid 3.1 μL, iso-valeric acid 3.1 μL, iso-butyric acid, 3.1 μL), or on EG agar medium (per 1 L; Lab-Lemco powder 2.4 g, proteose peptone 10 g, yeast extract 5 g, Na_2HPO_4 4 g, glucose 1.5 g, soluble starch 0.5 g, L-cystine 0.2 g, Tween 80 1 mL, agar 15 g, L-cysteine·HCl·H_2O 0.5 g, horse blood 50 mL). After incubation for 1 to 2 days at 37°C in anaerobic conditions, we selected approximately 4000 varied colonies on the plates followed by picking up and inoculating on YCFA agar medium or EG agar medium. To perform single colonization, we repeated the pick-up and reinoculations, and then stocked all isolates at −80°C with 20% glycerol. DNA was extracted from a portion of the isolated colonies just before freezing for preservation by bead-based homogenization in 5% Triton X-100. To identify the species of isolates, the 16S rRNA gene was amplified using the universal bacterial primers 27F (5′-AGAGTTTGATCCTGGCTCAG-3′)[71] and 1492R (5′-GGTTACCTTGTTACGACTT-3′).[72] PCR products were partially sequenced using the 27F primer, and then we identified the species of isolates based on 3 different Basic Local Alignment Search Tool (BLAST) databases (NCBI, Eztaxon and Living Tree Project, date: Nov 2016).

Phylogenetic analysis of F. prausnitzii

To obtain the full-length 16S rRNA gene, we performed sequencing of the 16S rRNA gene of the isolated *F. prausnitzii* strains using primers 27F, 1492R, 520F (5′-CAGGAGTGCCAGCAGCCGCGG-3′), 520R (5′-ACCGCGGCTGCTGGC-3′), 1100F (5′-CAGGAG CAACGAGCGCAACCC-3′), and 1100R (5′-AGGGTTGCGCTCGTTG-3′)[73] using the dye-terminator method. Each sequence obtained by the 6 different primers was assembled using GeneStudio (Professional Edition version 2.2.0.0). The similar available sequences,[74] representing close relatives of the isolates (sequence accession numbers are shown at the last part of the strain name in Figure S3), were retrieved from public databases (NCBI; date: Apr 2020). Evolutionary analyses were conducted using MEGA (version 10.1.7)[46] (Figure S3). Briefly, evolutionary history was inferred using the neighbor-joining method.[71] Tree topology was evaluated by a bootstrap analysis with 1000 replicates. The evolutionary distances were computed using the Kimura 2-parameter method.[71]

Preparation of bacterial samples

Bacterial samples for animal experiments were prepared using the following method. *F. prausnitzii* strains were first maintained for 24 hours at 37°C in YCFA liquid medium (YCFA agar medium excluding agar and added with resazurin 0.5 mg/L) and then transferred to LYBHI liquid medium (pre 1 L; brain heart infusion 37 g, yeast extract 5 g, hemin 5 mg, cellobiose 1 g, maltose·H_2O 1.1 g, resazurin 0.5 mg, L-cysteine·HCl·H_2O 0.7 g) at a ratio of 5%. After incubation for 24 or 48 hours at 37°C, bacterial cultures were processed by centrifugation at 800 g for 10 minutes at room temperature. To obtain live bacterial samples, we resuspended the bacterial pellets with culture media. This was to minimize the risk of repeated exposure to oxygen during washing and centrifugation on the survival of the strictly anaerobic live bacteria. The live bacterial solutions were stored at −80°C with 15% glycerol under anaerobic conditions until further use for the live bacterial groups of the animal experiments. The culture media as well as the live bacterial solutions were stored at −80°C with 15% glycerol under anaerobic conditions until further use for the medium-administered groups of the animal experiment. Except for centrifugation, all manipulations were performed in anaerobic chambers (N_2 = 90%, CO_2 = 5%, H_2 = 5%). Regarding the preparation of culture supernatant, the culture medium was centrifuged (800 g, room temperature, 10 minutes) and filtered (0.22 μm pore size) to obtain the culture supernatant. The culture supernatant was mixed with 15% glycerol and stored at −80°C until administration to the animals. Regarding the preparation of pasteurized bacteria, the culture medium was centrifuged (800 g, room temperature, 10 minutes) to remove the supernatant, and the bacterial pellets were washed twice with saline solution. The bacterial pellets were then resuspended in saline solution to achieve the same turbidity as that of the live bacterial group and then pasteurized (70°C, 30 minutes). The pasteurized bacterial solution was stored at −80°C until animal administration.

Intracerebroventricular (i.c.v.) injection

We used an Aβ-injected mouse model, of which validation was reported[10] based on three types of validity: face, construct, and predictive validity. Face validity was assessed based on the symptomatic results that the Aβ-injected mice significantly displayed impaired memory acquisition, but not memory retrieval, which implies the similar episodic anterograde memory deficiency observed in the early stages of human AD patients. Construct validity was assessed based on the physiological results that the Aβ-injected mice also displayed significantly attenuated hippocampal long-term potentiation. Predictive validity was assessed based on the results that

the treatment with galantamine, an approved drug for AD, significantly improved cognitive dysfunction in this model. The authors thus concluded that the Aβ-injected mouse model displays specific dysfunction of memory processes, which at least partly fulfills the three validity criteria for AD. Furthermore, similar experimental condition were also used as reported in other studies.[11–16] In these studies, Aβ25-35 has been widely used. This is because Aβ25-35 is generally considered as the biologically active region of Aβ, as introduced in several studies.[75–77] Regarding the dose of Aβ25-35, we used similar dose as used in these studies. Thus, there are symptomatic and pathophysiological similarities of this model with human AD and this validated animal model could be useful in evaluating the candidates of Alzheimer's-type dementia intervention through behavioral experiments. Aβ protein 25-35 (PolyPeptide Laboratories, USA) was dissolved in distilled water (final concentration 2 mM) and incubated at 37°C for 96 hours. I.c.v. injection was performed as described previously with some modifications.[68] Briefly, each mouse was anaesthetized by intraperitoneal injection of pentobarbital (40 mg/kg, Tokyo Chemical Industry Co., Ltd., Japan) and subcutaneous injection of 0.25% levobupivacaine (Maruishi Pharmaceutical Co.Ltd., Japan), and fixed in a stereotaxic frame (Narishige Inc., Japan). A burr hole was drilled in the skull at the following position: 0.2 mm posterior from bregma and 1 mm to the right of the midline. A 28-gauge needle connected by a syringe pump was inserted into the hole (2.5 mm depth from the brain surface), and Aβ25-35 solution (3 μL, 6 nmol) was then injected i.c.v. (1.0 μL/minutes). After the injection, the needle was held in place for an additional 3 minutes and then withdrawn.

Behavioral tests

To evaluate working memory, the Y-maze test was performed 6 days after the i.c.v. injection[68] (6 days of recovery is enough to get accurate behavior results as reported[10–16]) (Figures 3, 4, and S4). The maze was made of polyvinyl plastic and had three arms (395 mm depth, 120 mm height, 45 mm width at the bottom, and 100 mm width at the top), which were symmetrically disposed at 120° angles from each other. Mice were initially placed at the end of one arm and allowed to freely move for 7 minutes. The sequence of arm entries was manually counted to calculate the total number of entries and the alternation ratio (ratio of the number of alternations to (the total number of entries minus 2)). This test was performed by experimenters blind to the group assignments.

One day after the Y-maze test, the PA test was carried out for evaluating long-term memory with some modifications[78] (Figures 3, 4, and S4). The apparatus consisted of dark (240 mm width, 245 mm depth, 300 mm height) chamber with grid floors and a light (100 mm width, 100 mm depth, 300 mm height) chamber. The acquisition trial was started by placing each mouse in the light chamber. The guillotine door between the two chambers was opened after 10 s, and the initial latency time until entering into the dark chamber was recorded. When the mice had completely moved into the dark chamber, an electric foot shock (0.2 mA, 2 s duration, scrambled) was delivered to the mice. Afterward, the mice were placed back into their original mice cage. Twenty-four hours after the acquisition trial, the mice were placed in the light chamber again, and the latency time until entering into the dark chamber was measured with a cut-off time of 300 s as the test trial.

Whole-genome comparison

To obtain genomic DNA of *F. prausnitzii* strains for PacBio sequencing, *F. prausnitzii* strains were first incubated for 24 hours at 37°C in YCFA liquid medium and then transferred to LYBHI liquid medium at a concentration of 5%. After incubation for 24 or 48 hours at 37°C, bacterial cultures were processed by centrifugation at 800 g for 10 minutes at 4°C. The bacterial pellets were washed with PBS supplemented with 0.5 g L-cysteine·HCl·H_2O and 1 mg resazurin followed by centrifugation at 800 g for 10 minutes at 4°C. This washing was repeated twice for a total of three PBS washes. The bacterial pellets were stored at −80°C until use. Genomic DNA was extracted using the NucleoBond AXG 20 and NucleoBond Buffer Set III (Macherey-nagel GmbH & Co., Germany) and fragmented by g-TUBE™ (Covaris, Inc., USA) according to the manufacturer's protocol. Extracted DNA samples were sequenced using PacBio RS II (Pacific Biosciences, USA). The obtained reads were assembled *de novo* using PacBio SMRT Analysis (v2.3). Circlator (v1.2.1)[47] was used for circularizing genome assemblies. After circularization, Quiver was used for polishing. The genome completeness and contamination were determined using CheckM (v1.0.08) (Table S2).[48]

CDS prediction and genome annotation were performed using Prokka (v1.12).[49] Protein domains were predicted using InterProScan (v5.40-77.0)[50] and TMHMM (v2.0).[51] Regarding TMHMM, if the whole sequence is labeled with inside or outside, the prediction is that it contains no membrane and is regarded as either an intracellular protein or an extracellular protein. If the whole sequence is labeled with at least one transmembrane helix, the prediction is that it is regarded as a transmembrane protein. Genes were annotated with KEGG genes and KEGG Orthologs (KOs, date: May 2017) using DIAMOND (v0.9.10.111)[54] with thresholds of identity > 40, score > 70, and coverage > 80. Orthologs were identified by using two different ortholog finding tools, Roary (v3.12.0)[52] and Orthofinder (v2.3.11),[53] to enhance the precision of the analysis. KOs and domain profiles were assigned to each ortholog. Orthologs found only in specific strains or orthologs that contained KOs found only in specific strains were defined as "specific orthologs." The pipeline for whole-genome comparison is shown in Figure S5. The results of ortholog, domain, and KO assignment are summarized in Table S5.

Regarding the whole genome comparison between known strains of *F. prausnitzii* and our effective strains, Fp14 and Fp360, we used the complete genome of three strains (A2-165: GCA_010509575.1, S3L/3: GCA_000209855.1 and L2-6: GCA_000210735.1) among 8 strains of *F. prausnitzii* in Figure S3, which were available in NCBI. The genome of the remaining five were either not deposited or represented only by draft assemblies (https://www.ncbi.nlm.nih.gov/genome/genomes/682; date: Apr 2021).

Metagenome shotgun sequencing

Sequencing libraries were generated with a TruSeq Nano DNA Library Prep Kit (Illumina). Library quality was confirmed with an Agilent 2100 BioAnalyzer. Metagenome shotgun sequencing of fecal samples was carried out on the HiSeq2500 platform (Illumina). All samples were paired-end sequenced with a 150-bp read length to a targeted data size of 15.0 Gb.

Quality control of whole-genome shotgun reads

A total of 6,421,909,374 (149,346,730 on average) paired-end reads, covering 963,286,406,100 (22,402,009,444 on average) base pairs in total, underwent quality control as follows (Figure S5; Table S3).[69] Raw reads containing the letter 'N' (base pair not identified) were discarded. Reads containing the bacteriophage *PhiX* DNA sequences were identified by mapping them against the reads using Bowtie 2 (v2.2.9)[56] with preset options in '–fast-local' and discarded. Reads were trimmed for adaptor sequences and primer sequences using Cutadapt (v1.9.1),[55] for which the following options were used: '-a AGATCGGAAGAGCACACGTCTGAACTC CAGTCA -O 32 -q 17' for the forward primer sequence; '-a AGATCGGAAGAGCGTCGTGTAGGGAAAGAGTGT -O 32 -q 17' for the reverse primer sequence. Reads consecutively containing quality values of 17 or less were tailed-cut at the 3′ termini with the Cutadapt program. Next, reads of lengths less than 50 base pairs were discarded. Reads with average quality values of 25 or less were discarded. Next, reads were mapped against the human genome (24 gi numbers: from 568336000 to 568336023; http://www.ncbi.nlm.nih.gov/nuccore/568336023/; GRCh38) using Bowtie2. Those that were mapped were considered to be derived from the human genome and were discarded. Finally, unpaired reads were discarded. As a result, a total of 6,229,896,106 (144,881,305 on average) paired-end reads with 934,484,415,900 (21,732,195,719 on average) base pairs in total (referred to as the 'high-quality reads' hereafter) were used for the following analyses (Figure S5; Table S3).

Calculation of ortholog abundance

The abundance of each gene from the isolated strains was evaluated based on transcripts per million (TPM) as calculated by Kallisto (v0.46.2).[57] Each gene was categorized into an ortholog group, which was identified by Roary or Orthofinder, and the abundance of the orthologs was calculated (Figures 6 and S5; Table S6).

Metabolome Analysis

Approximately 40-50 mg of frozen hippocampus was placed in a homogenization tube, along with zirconia beads (5mmφ and 3mmφ). Next, 50% acetonitrile/Milli-Q water containing internal standards (H3304-1002, Human Metabolome Technologies, Inc. (HMT), Japan) was added to the tube, after which the tissue was completely homogenized at 1,100 rpm, 4°C for 120 s twice using a beads shaker (Shake Master NEO, Bio Medical Science, Japan). The homogenate was then centrifuged at 2,300 × *g*, 4°C for 5 min. Subsequently, 400 μL of upper aqueous layer was centrifugally filtered through a Millipore 5-kDa cutoff filter (UltrafreeMC-PLHCC, HMT) at 9,100 × *g*, 4°C for 120 min to remove macromolecules. The filtrate was evaporated to dryness under vacuum and reconstituted in 50 μL of Milli-Q water for metabolome analysis at HMT.

Metabolome analysis was conducted using capillary electrophoresis Fourier transform mass spectrometry (CE-FTMS). CE-FTMS analysis was carried out using an Agilent 7100 CE capillary electrophoresis system equipped with a Q Exactive Plus (Thermo Fisher Scientific Inc., USA), Agilent 1260 isocratic HPLC pump, Agilent G1603A CE-MS adaptor kit, and Agilent G1607A CE-ESI-MS sprayer kit (Agilent Technologies, Inc., USA). The systems were controlled by Agilent MassHunter workstation software LC/MS data acquisition for 6200 series TOF/6500 series Q-TOF version B.08.00 (Agilent Technologies) and Xcalibur (Thermo Fisher Scientific), and connected by a fused silica capillary (50 μm *i.d.* × 80 cm total length) with commercial electrophoresis buffer (H3301-1001 and I3302-1023 for cation and anion analyses, respectively, HMT) as the electrolyte. The MS-spectrometer was scanned from m/z 60 to 900 (cation) and 70 −1050 (anion). Peaks were extracted using MasterHands, automatic integration software (Keio University, Japan) in order to obtain peak information including *m/z*, peak area, and migration time (MT).[79] Signal peaks corresponding to isotopomers, adduct ions, and other product ions of known metabolites were excluded, and the remaining peaks were annotated according to HMT's metabolite database based on their *m/z* values and MTs. Areas of the annotated peaks were then normalized to internal standards and sample volume in order to obtain relative levels of each metabolite (Figure 7; Table S7).

RNA-seq Analysis

Total RNA was extracted form mouse hippocampus by the RNeasy® Plus Universal Mini Kit (QIAGEN, Netherlands) according to the manufacturer's instructions. Sequencing libraries were generated with a TruSeq Stranded mRNA Library Prep Kit (Illumina). Library quality was confirmed with an Agilent 2100 BioAnalyzer. RNA sequencing was carried out on the NovaSeq 6000 platform (Illumina). All samples were paired-end sequenced with a 150-bp read.

Raw reads containing the letter 'N' (base pair not identified) were discarded. Reads containing the bacteriophage *PhiX* DNA sequences were identified by mapping them against the reads using Bowtie 2 (v2.2.9)[56] with preset options in '–fast-local' and discarded. Reads were trimmed for adaptor sequences and primer sequences using Cutadapt (v1.9.1),[55] for which the following options were used: '-a GATCGGAAGAGCACACGTCTGAACTCCAGTCAC -O 33 -q 17' for the forward primer sequence; '-a AGATCGGAA GAGCGTCGTGTAGGGAAAGAGTGT -O 32 -q 17' for the reverse primer sequence. Reads consecutively containing quality values of 17 or less were tailed-cut at the 3′ termini with the Cutadapt program. Next, reads of lengths less than 50 base pairs were discarded. Reads with average quality values of 25 or less were discarded. Finally, unpaired reads were discarded.

Next, transcript-level expression analysis was performed as previously described with some modification.[80] Reads were mapped against the *Mus musculus* genome GRCm38.p6 by HISAT2.[60,58] Transcript quantification was performed by StringTie.[59] Differential expression analysis was performed by DESeq2 using R (v3.6.3). We performed three independent RNA-seq experiments. Transcripts, which were determined to be differentially expressed in more than two experiments, were selected as differentially expressed transcripts (Table S8).

QUANTIFICATION AND STATISTICAL ANALYSIS

The abundance of bacterial genera and orthologs, within-group Bray-Curtis distance, characteristics of the selected subjects and Shannon-Wiener alpha-diversity index were determined to be either significantly increased or decreased in each stage (MCI and AD) compared with the healthy group using a two-sided Wilcoxon rank sum test of R (v3.6.3) (Figures 2, S2, and 6; Tables S1, S4, and S6). Permutational multi-variate analysis of variance (PERMANOVA) by R (v3.6.3) was applied to test microbial composition between groups. In addition, a Benjamini-Hochberg false-discovery rate-corrected p value (q value) was estimated. We evaluated the relationship between *Faecalibacterium* and cognitive function found in the cross-sectional study by the animal experiments. The behavioral responses of the administered groups were determined to be significantly improved by a comparison with the vehicle group or the medium-administered group using a two-sided unpaired Student's t test of SAS software (v9.4) (Figures 3, 4, and S4). The metabolites were determined to be significantly changed by a comparison with the vehicle group using a two-sided unpaired Welch's t test by R (v3.6.3) (Figure 7; Table S7). Transcripts were determined to be differentially expressed by a comparison with the vehicle group using the Wald test along with a correction of Benjamini-Hochberg false-discovery rate (cut-off value was 0.1) by R (v3.6.3) (Table S8). Correlations between the relative abundance of bacterial genera and MoCA-J scores or age were calculated by Spearman's rank-correlation analysis using R (v3.6.3) (Figures 2, S2, and Table S4). $p < 0.05$ was considered statistically significant.

Cell Reports Medicine, Volume *2*

Supplemental information

Identification of *Faecalibacterium prausnitzii* strains for gut microbiome-based intervention in Alzheimer's-type dementia

Atsushi Ueda, Shoji Shinkai, Hirotsugu Shiroma, Yu Taniguchi, Sayaka Tsuchida, Takahiro Kariya, Tomohiro Kawahara, Yodai Kobayashi, Noriyuki Kohda, Kazunari Ushida, Akihiko Kitamura, and Takuji Yamada

Supplemental information:

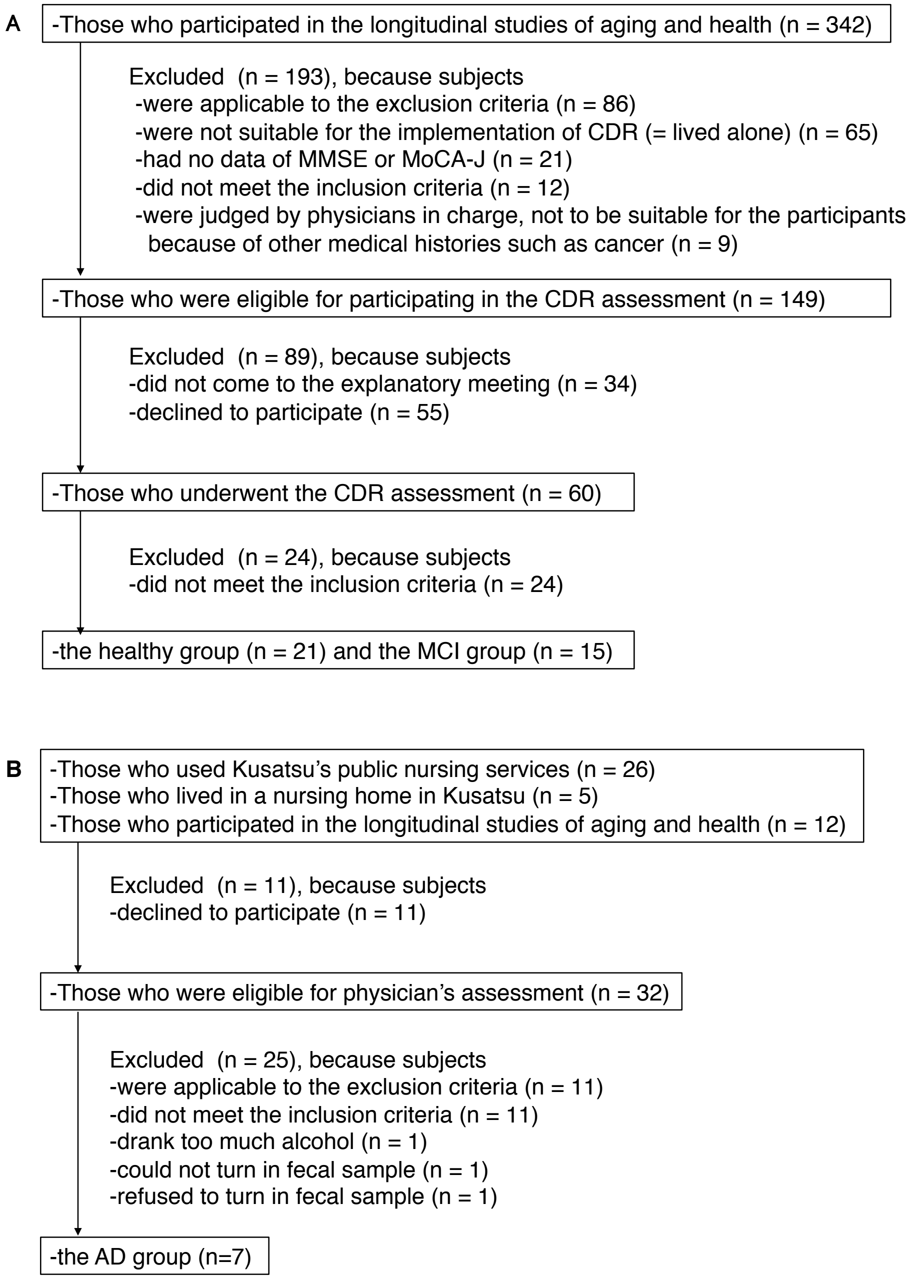

Figure S1. Flow diagram of the selection of the subjects. Related to Figure 2.

(A) Flow diagram showing the selection of subjects in the healthy group and the MCI group. (B) Flow diagram showing the selection of subjects in the AD group.

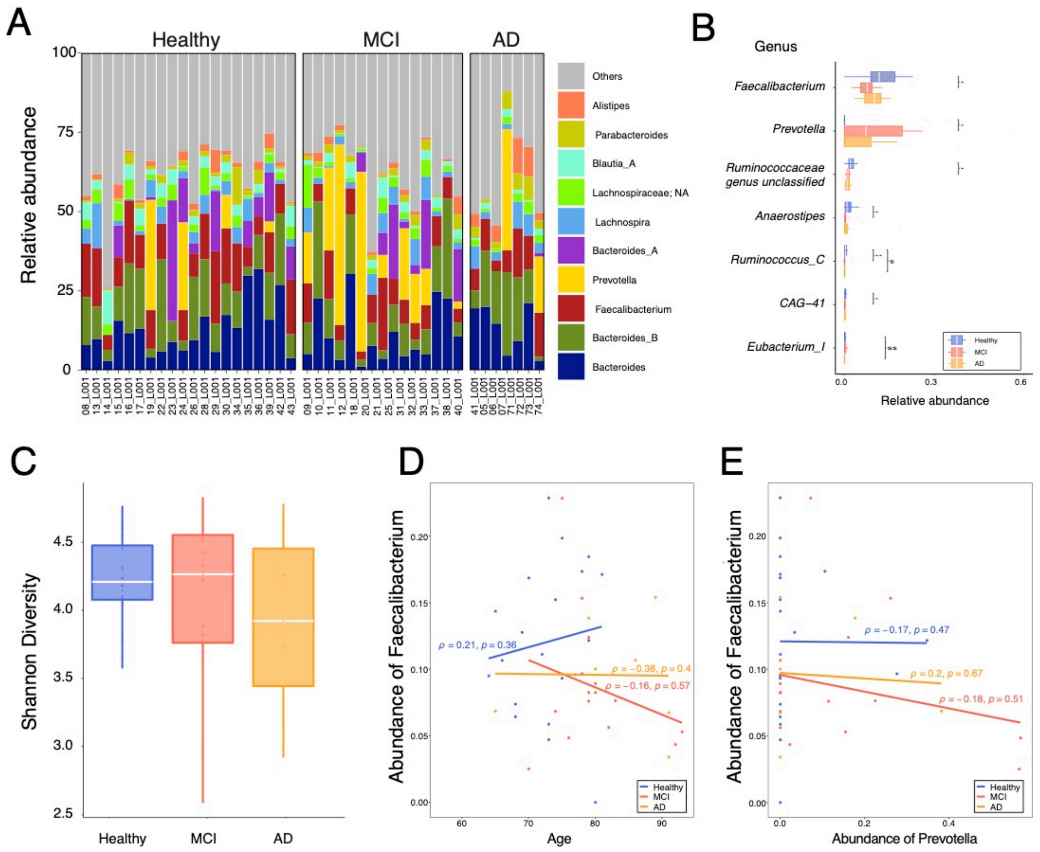

Figure S2. Plot showing the relative abundance of genera, alpha-diversity, and the correlation between the relative abundance of *F. prausnitzii* and age or the relative abundance of *Prevotella*. Related to Figure 2 and Table S4.

(A) Stacked bar plot showing the overall genus composition in each individual. (B) Box plot showing the interquartile range (IQR) of the relative abundance of genera. Seven genera whose abundance was significantly different either between the healthy (n=20) and the MCI group (n=15) or between the healthy and the AD group (n=7) are shown (Wilcoxon rank sum test, the healthy vs the MCI: $^{**}p < 0.01$, $^{*}p < 0.05$, the healthy vs the AD: $^{\$\$}p < 0.01$, $^{\$}p < 0.05$). (C) Box plot showing the IQR of the Shannon-Wiener alpha-diversity index, which did not differ between groups. (D, E) Scatter plot showing Spearman's ρ and p value between the relative abundance of *F. prausnitzii* and age or the relative abundance of *Prevotella* of the healthy (blue), the MCI (red), and the AD group (orange). There was no significant correlation between the abundance of *Faecalibacterium* and age or the abundance of *Prevotella*.

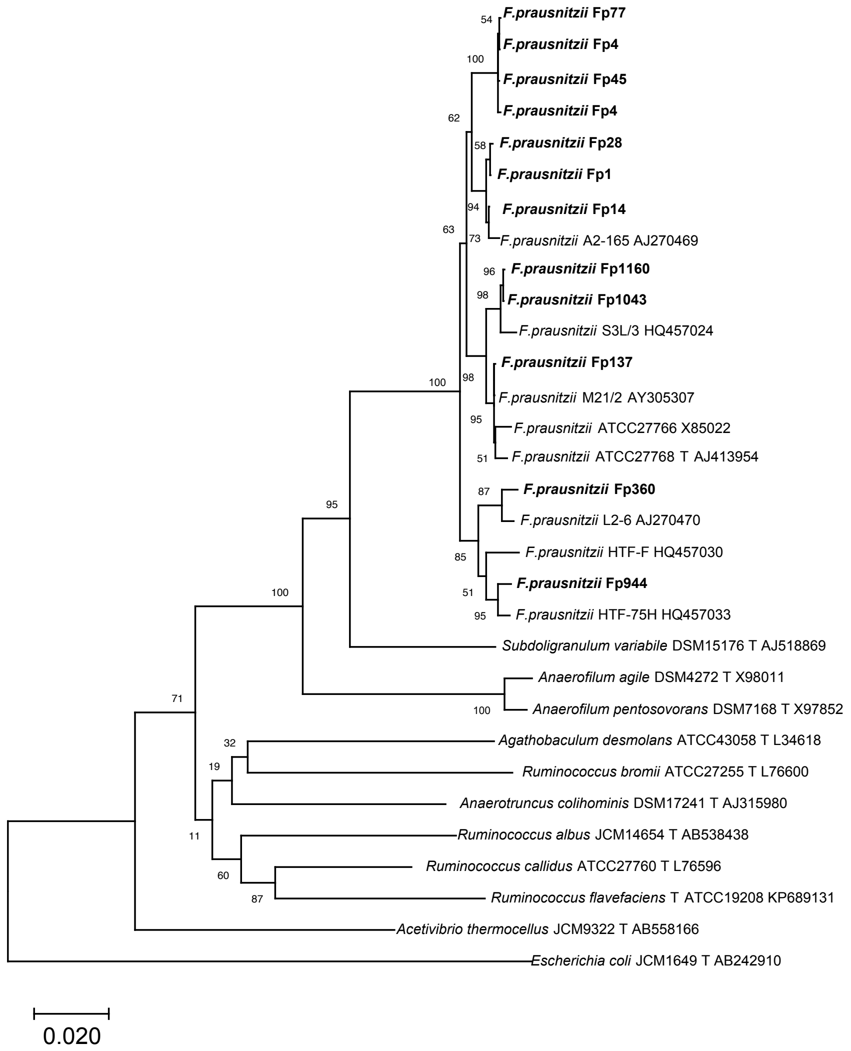

Figure S3. *F. prausnitzii* strains were isolated as candidates for gut microbiome-based intervention in MCI. Related to Figure 2.

Phylogenetic tree showing the relationships of the 12 *F. prausnitzii* isolates with other members of *Ruminococcaceae* based on 16S rRNA gene sequences. A neighbor-joining phylogenetic tree was constructed using MEGA. Bootstrap values (expressed as a percentage of 1,000 replications) are shown at branching points. The scale bar indicates the number of substitutions per nucleotide position. *F. prausnitzii* isolates in this study are highlighted in bold. Sequence accession numbers are shown at the last part of the strain name. “T” is the abbreviation for “type strain”.

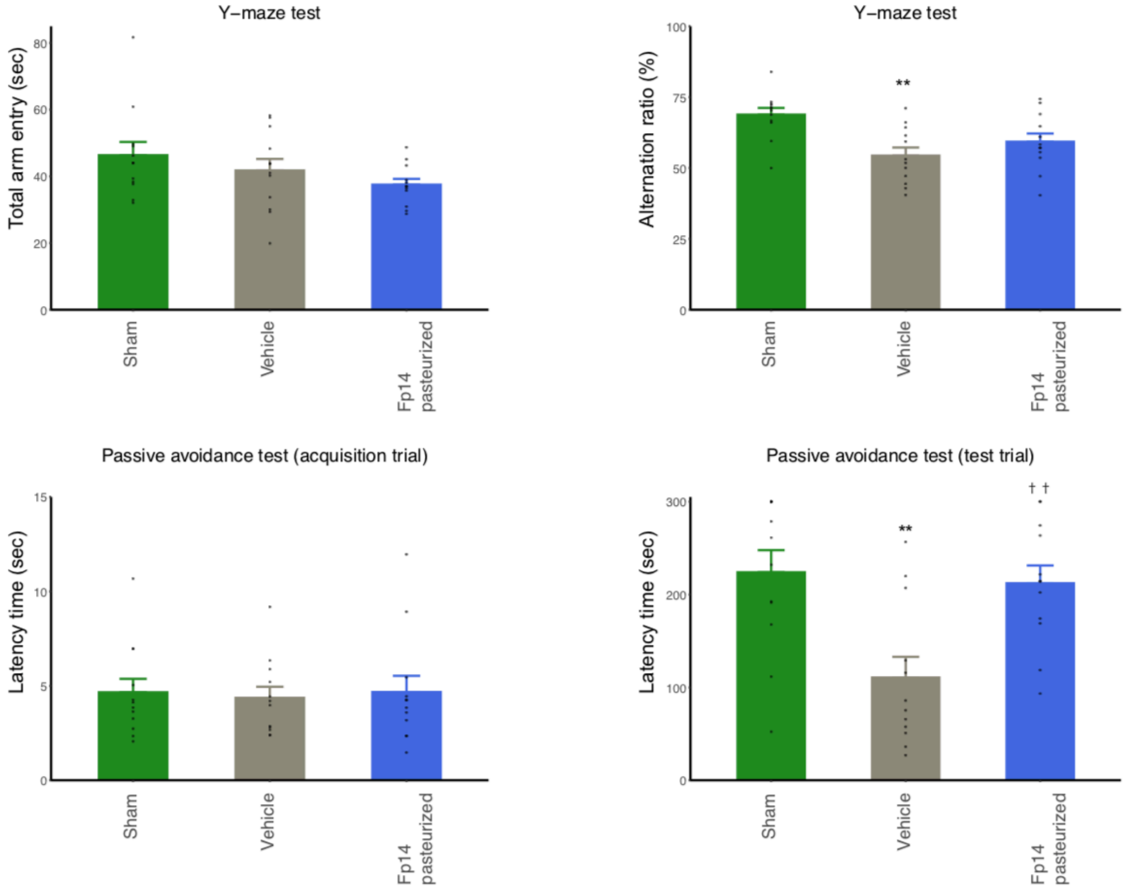

Figure S4. Pasteurized Fp14 reproducibly improved Aβ-induced cognitive impairment. Related to Figure 4.

Pasteurized Fp14 were orally administered to mice injected i.c.v. with Aβ25-35. Cognitive performance was evaluated by the Y-maze test and the passive avoidance test. (A) Bar plot showing the total entry time in the Y-maze test. (B) Bar plot showing the alternation ratio in the Y-maze test. (C) Bar plot showing the latency time of the acquisition trial in the passive avoidance test. (D) Bar plot showing the latency time of the test trial in the passive avoidance test. All values are expressed as the mean + S.E. (n=12, biological replicates). $^{**}p < 0.01$ by two-sided unpaired Student's *t*-test (sham-operation vs vehicle); $^{††}p < 0.01$ by two-sided unpaired Student's *t*-test (vehicle vs Fp14 pasteurized).

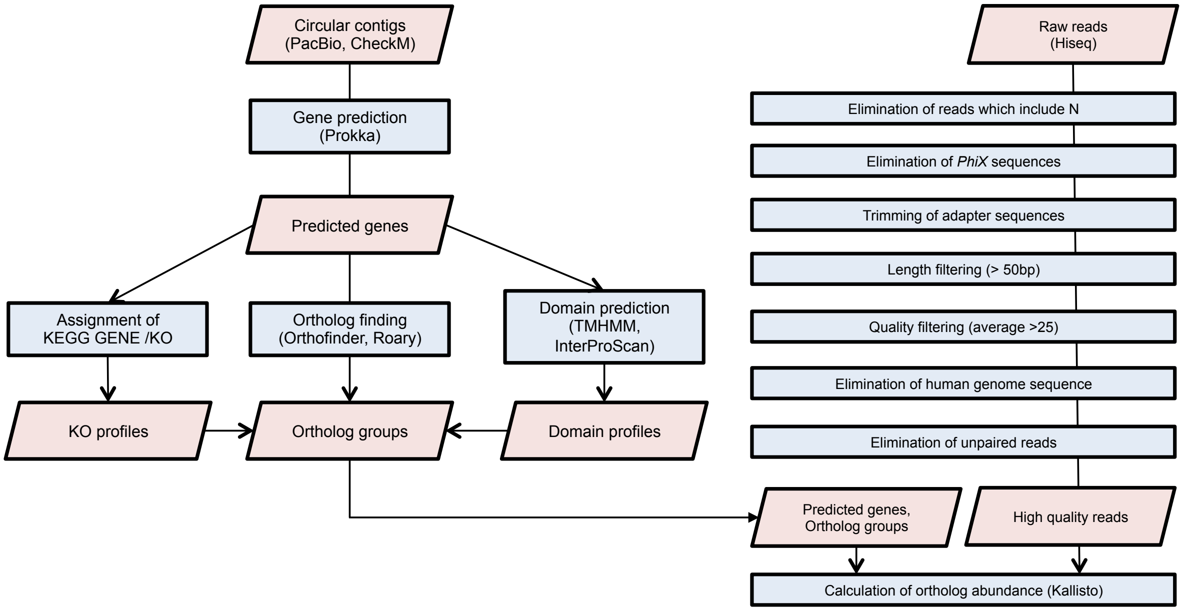

Figure S5. Pipeline for whole-genome comparison and whole-metagenome shotgun sequencing analysis. Related to Figure 5 and 6.

Regarding the whole-genome comparison, circular contigs from Pacbio underwent a quality control check by CheckM and gene prediction by Prokka. Protein domains were predicted using InterProScan, and TMHMM. Genes were annotated with KEGG genes and KEGG Orthologs (KOs) using DIAMOND. Orthologs were identified by using two different ortholog finding tools, Roary and Orthofinder, to enhance the precision of the analysis. KOs and domain profiles were assigned to each ortholog. Regarding the whole-metagenome shotgun sequencing analysis, raw reads first underwent a quality control to generate high-quality reads. The abundance of each gene from the isolated strains was evaluated by Kallisto. Each gene was categorized into an ortholog group, which was identified by Roary or Orthofinder, and the abundance of the orthologs was calculated (see also Methods Details).

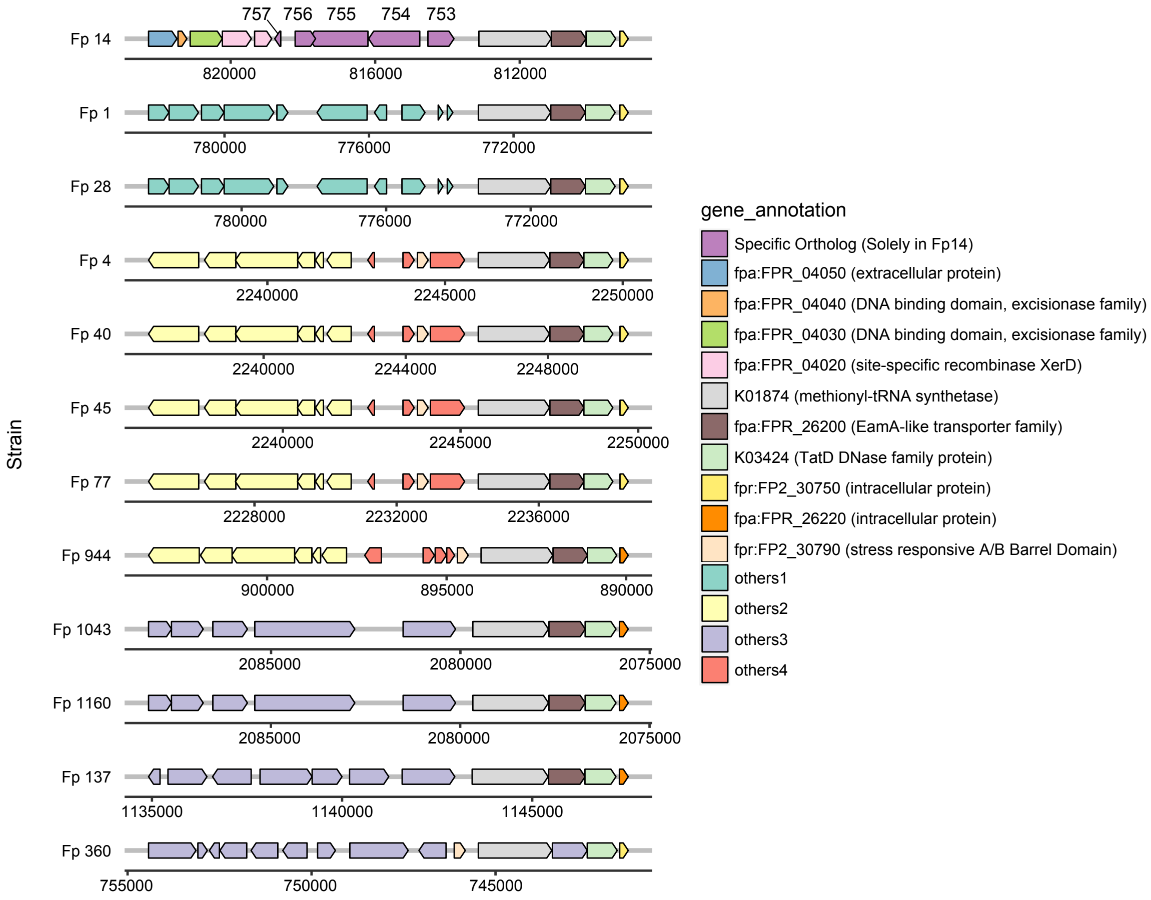

Figure S6. Whole-genome comparison revealed specific orthologs in the Fp14. Related to Figure 5.

Complete genomes of the 12 *F. prausnitzii* isolates were obtained using a PacBio sequencer. Specific orthologs were identified by using different ortholog finding tools, Roary and Orthofinder. Orthologs found only in specific strains or orthologs that contained KEGG Orthologs (KOs) found only in specific strains were defined as “specific orthologs”. KOs and domain profiles were assigned to each ortholog using DIAMOND, InterProScan, and TMHMM. 5 of 150 specific orthologs found only in Fp14 are shown.

Table S1. The characteristics of the selected subjects. Related to Figure 2 and 6.

Table showing the characteristics of the selected subjects in this study. All values except gender are expressed as the mean ± S.D. *P* value except for gender was calculated by Wilcoxon rank sum test (vs the healthy group). *P* value for gender was calculated by Fisher's exact test (vs the healthy group).

Number	Healthy (n = 21)	MCI (n = 15)	AD (n = 7)	*p* value (HvsM)	*p* value (HvsA)
Age (years)	72.7±5.3	79.8±6.1	83.0±9.3	0.0014	0.0092
Gender (n, % Female)	13, 61.9	9, 60.0	5, 71.4	1	1
BMI (kg/m^2)	22.7±3.7	24.4±2.3	22.9±2.7	0.1828	0.8735
MMSE (score)	29.5±0.7	26.9±2.3	15.6±4.3	0.0001	<0.0001
MoCA-J (score)	27.6±1.5	19.8±2.7	8.4±4.0	<0.0001	<0.0001
Education (years)	11.2±1.7	9.8±3.6	8.3±2.0	0.2146	0.0025

Table S2. The completeness and the contamination of the genomes of the 12 isolated *F. prausnitzii*. Related to Figure 5.

All genomes were estimated to be ≥95% complete with ≤5% contamination by CheckM, making them excellent data sets for whole-genome comparison(Parks et al., 2015).

Strain	Completeness (%)	Contamination (%)	Strain heterogeneity (%)
Fp1	100	0	0
Fp4	100	0	0
Fp14	100	0	0
Fp28	100	0	0
Fp40	100	0	0
Fp45	100	0	0
Fp77	100	0	0
Fp137	100	0	0
Fp360	100	0	0
Fp944	100	0	0
Fp1043	100	0	0
Fp1160	100	0.34	100
Fp1233	100	0	0
